# Comparison of methods for predicting COVID-19-related death in the general population using the OpenSAFELY platform

**DOI:** 10.1186/s41512-022-00120-2

**Published:** 2022-02-24

**Authors:** The OpenSAFELY Collaborative, Elizabeth J. Williamson, John Tazare, Krishnan Bhaskaran, Helen I. McDonald, Alex J. Walker, Laurie Tomlinson, Kevin Wing, Sebastian Bacon, Chris Bates, Helen J. Curtis, Harriet J. Forbes, Caroline Minassian, Caroline E. Morton, Emily Nightingale, Amir Mehrkar, David Evans, Brian D. Nicholson, David A. Leon, Peter Inglesby, Brian MacKenna, Nicholas G. Davies, Nicholas J. DeVito, Henry Drysdale, Jonathan Cockburn, William J. Hulme, Jessica Morley, Ian Douglas, Christopher T. Rentsch, Rohini Mathur, Angel Wong, Anna Schultze, Richard Croker, John Parry, Frank Hester, Sam Harper, Richard Grieve, David A. Harrison, Ewout W. Steyerberg, Rosalind M. Eggo, Karla Diaz-Ordaz, Ruth Keogh, Stephen J. W. Evans, Liam Smeeth, Ben Goldacre

**Affiliations:** 1 London School of Hygiene and Tropical Medicine, Faculty of Epidemiology & Population Health, Keppel Street, London, WC1E 7HT UK; 2 NIHR Health Protection Research Unit (HPRU) in Immunisation, London, UK; 3 The DataLab, Nuffield Department of Primary Care Health Sciences, University of Oxford, Oxford, OX26GG UK; 4 TPP, TPP House, 129 Low Lane, Horsforth, Leeds, LS18 5PX UK; 5 University of Bristol, Beacon House, Queens Road, Bristol, BS8 1QU UK; 6 Intensive Care National Audit & Research Centre (ICNARC), 24 High Holborn, Holborn, London, WC1V 6AZ UK; 7 Leiden University Medical Center, Leiden, the Netherlands

**Keywords:** Risk prediction, Risk stratification, Mortality, COVID-19, Infectious disease, Statistical methodology

## Abstract

**Background:**

Obtaining accurate estimates of the risk of COVID-19-related death in the general population is challenging in the context of changing levels of circulating infection.

**Methods:**

We propose a modelling approach to predict 28-day COVID-19-related death which explicitly accounts for COVID-19 infection prevalence using a series of sub-studies from new landmark times incorporating time-updating proxy measures of COVID-19 infection prevalence. This was compared with an approach ignoring infection prevalence.

The target population was adults registered at a general practice in England in March 2020. The outcome was 28-day COVID-19-related death. Predictors included demographic characteristics and comorbidities. Three proxies of local infection prevalence were used: model-based estimates, rate of COVID-19-related attendances in emergency care, and rate of suspected COVID-19 cases in primary care.

We used data within the TPP SystmOne electronic health record system linked to Office for National Statistics mortality data, using the OpenSAFELY platform, working on behalf of NHS England.

Prediction models were developed in case-cohort samples with a 100-day follow-up. Validation was undertaken in 28-day cohorts from the target population. We considered predictive performance (discrimination and calibration) in geographical and temporal subsets of data not used in developing the risk prediction models. Simple models were contrasted to models including a full range of predictors.

**Results:**

Prediction models were developed on 11,972,947 individuals, of whom 7999 experienced COVID-19-related death. All models discriminated well between individuals who did and did not experience the outcome, including simple models adjusting only for basic demographics and number of comorbidities: C-statistics 0.92–0.94. However, absolute risk estimates were substantially miscalibrated when infection prevalence was not explicitly modelled.

**Conclusions:**

Our proposed models allow absolute risk estimation in the context of changing infection prevalence but predictive performance is sensitive to the proxy for infection prevalence. Simple models can provide excellent discrimination and may simplify implementation of risk prediction tools.

**Supplementary Information:**

The online version contains supplementary material available at 10.1186/s41512-022-00120-2.

## Key messages

- Evolving policies regarding restrictions on social contact and return to work require good information regarding risks of severe COVID-19 outcomes in the general population; however, obtaining accurate risk estimates is challenging in the context of changing levels of circulating infection, which standard risk prediction models commonly ignore.
- We describe methods for risk prediction adopting a landmarking approach to dynamically incorporate time- and region-dependent information on infection prevalence.
- We compare the discrimination and calibration of models that do and do not incorporate changing infection prevalence over time in different geographical and temporal settings, and compare simpler models to more richly specified ones.
- Our study suggests that models that ignore the infection prevalence provide poorly calibrated estimates of absolute risk; models that include time-varying measures of the infection prevalence can provide more accurate estimates.
- Simple models based only on a number of comorbidities and basic demographics performed almost as well as more complex risk prediction models, both within models including infection prevalence and models that ignore this, suggesting that policies targeting population-level reduction of COVID-19 mortality risk may not need to distinguish between all comorbidities in detail.

## Introduction

Characterised as a pandemic by the World Health Organization on 11 March 2020 [1], globally, the cumulative number of cases of COVID-19 has exceeded 180 million with almost 4 million deaths attributed to the virus at the time of writing [2]. Evolving policies such as return-to-work strategies and restrictions on social contact are heavily informed by the estimated risk of severe outcomes from COVID-19. Policy-making is often informed by absolute risks in the general population. This risk reflects the result of two processes: being infected and dying once infected, thus depends critically on infection prevalence. Transporting estimates of absolute risk from one context to another, such as a different time period or geographical region, is particularly challenging in COVID-19 due to substantial variation in the infection prevalence over time and by geography [3].

Prediction models that do not explicitly model the underlying infection prevalence may provide inaccurate estimates of absolute risk. Whether such models produce transportable rankings of risk, to different temporal and geographical contexts, is uncertain. Some predictors identified in these models, such as geographical region and characteristics that differ substantially by region, may be indirectly capturing differences in infection prevalence; models explicitly including the infection prevalence may allow simpler predictive models with equally good performance.

Incorporating the underlying infection prevalence is not straightforward because direct estimates are typically not available within small geographical areas. Whether easily accessible proxy measures are sufficiently accurate to produce reasonable risk estimates remains uncertain.

In this study, we compare modelling strategies to predict 28-day COVID-19 death which do and do not allow for dynamic predictions and illustrate the impact that these methodological choices have, in terms of model discrimination and calibration, using data from the first wave of COVID-19 in England held in the OpenSAFELY platform [4] on almost 12 million adults in England. Our overarching goal is to inform subsequent development of risk prediction models in this context, rather than to develop a risk prediction model to inform policy-making. Specifically, we aim to answer the following: (1) Do risk prediction models which do not explicitly model the underlying infection prevalence perform well in different geographical and temporal contexts? (2) Can transportable estimates of absolute risk of COVID-19 death be obtained by explicitly incorporating proxy estimates of the changing infection prevalence? (3) Can simpler prediction algorithms be used for predicting risk without losing substantial predictive ability, compared with more richly specified models?

## Methods

Our statistical approach is based on a model for 28-day COVID-19-related death detailed in the Supplementary Appendix. Our reporting adheres to the TRIPOD statement for reporting of multivariable prediction models [5].

### Data

#### Study population

The target population is adults in England living in the community; residential settings are excluded since risks experienced in institutions such as care homes are likely to be very different to those in smaller households.

#### Data source

Primary care records managed by the GP software provider TPP were linked to Office for National Statistics (ONS) death data through OpenSAFELY, a data analytics platform created by our team on behalf of NHS England to address urgent COVID-19 research questions (https://opensafely.org). OpenSAFELY provides a secure software interface allowing the analysis of pseudonymised primary care patient records from England in near real-time within the electronic health record vendor’s highly secure data centre, avoiding the need for large volumes of potentially disclosive pseudonymised patient data to be transferred off-site. This, in addition to other technical and organisational controls, minimises any risk of re-identification. Similarly, pseudonymised datasets from other data providers are securely provided to the electronic health record vendor and linked to the primary care data. Data includes pseudonymised data such as coded diagnoses, medications, and physiological parameters. No free text data are included.

#### Base cohort

A base cohort was defined, comprising males and females aged 18 years or older registered as of 1 March 2020 in a general practice employing the TPP system, followed up for 100 days (1 March 2020 until 8 June 2020). Individuals with missing age, sex, postcode, ethnicity, a recorded age over 105 years, or living in households of >10 people were excluded.

#### Outcome

The outcome was COVID-19-related death, defined by ICD-10 codes U07.1 or U07.2 anywhere on the death certificate.

#### Predictor variables

We selected candidate predictors based on known or plausible associations with exposure to COVID-19 infection, risk of severe illness or respiratory tract infection, and factors associated with healthcare access or level of care. Potential predictors were age, sex, ethnicity, deprivation, number in household, presence of young children in household, a rural indicator, obesity, smoking, blood pressure, and comorbidities (details in Supplementary Appendix).

Three different proxy measures of COVID-19 infection prevalence, measured daily, were available: (1) model-based estimates [6] available by region and age group, (2) rate of COVID-19-related A&E (emergency) attendances over the last week by local geographic area, and (3) rate of suspected COVID-19 cases over the last week by local geographic area (details in Supplementary Appendix).

### Development of risk prediction algorithms

Our goal is to predict the risk of 28-day COVID-19 death. Note that this is not death within 28 days of infection, but the risk of experiencing COVID-19 death within a 28-day period for individuals in our target population. Two approaches were adopted: approach A—models which do not explicitly account for the time-changing prevalence of COVID-19 infection, and approach B—landmarking models [7], which use a series of sequential overlapping 28-day sub-studies incorporating time-updating proxy measures of infection prevalence. Risk prediction models were developed using data from the base cohort. Case-cohort sampling was required to enable model fitting for approach B (the stacked sub-studies contain nearly 900 million rows of data). To reduce computational burden and increase comparability between approaches, case-cohort sampling was used to fit models for both approaches.

To develop models within approach A, follow-up began 1 March 2020 and ended at the first of COVID-19-related death or 8 June 2020. The outcome was COVID-19-related death (any time during the 100-day follow-up). No censoring was applied at death due to non-COVID causes, to target the sub-distribution hazard [8]; no other censoring events occurred. The analysis sample included all cases of COVID-19-related death and a random age-stratified sample of the base cohort (the ‘sub-cohort’) [9, 10], with sampling fractions of 0.01 in the age group 18–<40, 0.02 in 40–<60, 0.025 in 60–<70, 0.05 in 70–<80 and 0.13 in 80+ years.

Four approach A models were fitted, using different sets of predictor variables. The first used penalised regression (lasso) [11] to select a parsimonious model (the “selected” model; models were subsequently re-fitted including only the selected variables). The second included age group (10-category), sex and their interaction (“age-sex” model). The third additionally included grouped number of comorbidities (0, 1, 2, 3+), ethnicity and rural/urban (“comorbidities” model). The fourth included all 36 potential predictors and all possible interactions with age and sex (“full” model, details in the Supplementary Appendix).

Cox proportional hazards model were fitted including the relevant predictors using time in study as the timescale with Barlow weights to account for the case-cohort design and robust standard errors [9, 10]. The baseline survivor function was estimated at day 28 (${\hat{S}}_{28}$) and the estimated log hazard ratios ($\hat{\beta}$) were extracted. For an individual with predictor values *x*_*i*_, the predicted risk is then given by [12]: ${p}_i=1-{\hat{\ S}}_{28}^{\exp \left({\hat{\beta}}^T{x}_i\right)}$.

**Fig. 1:**
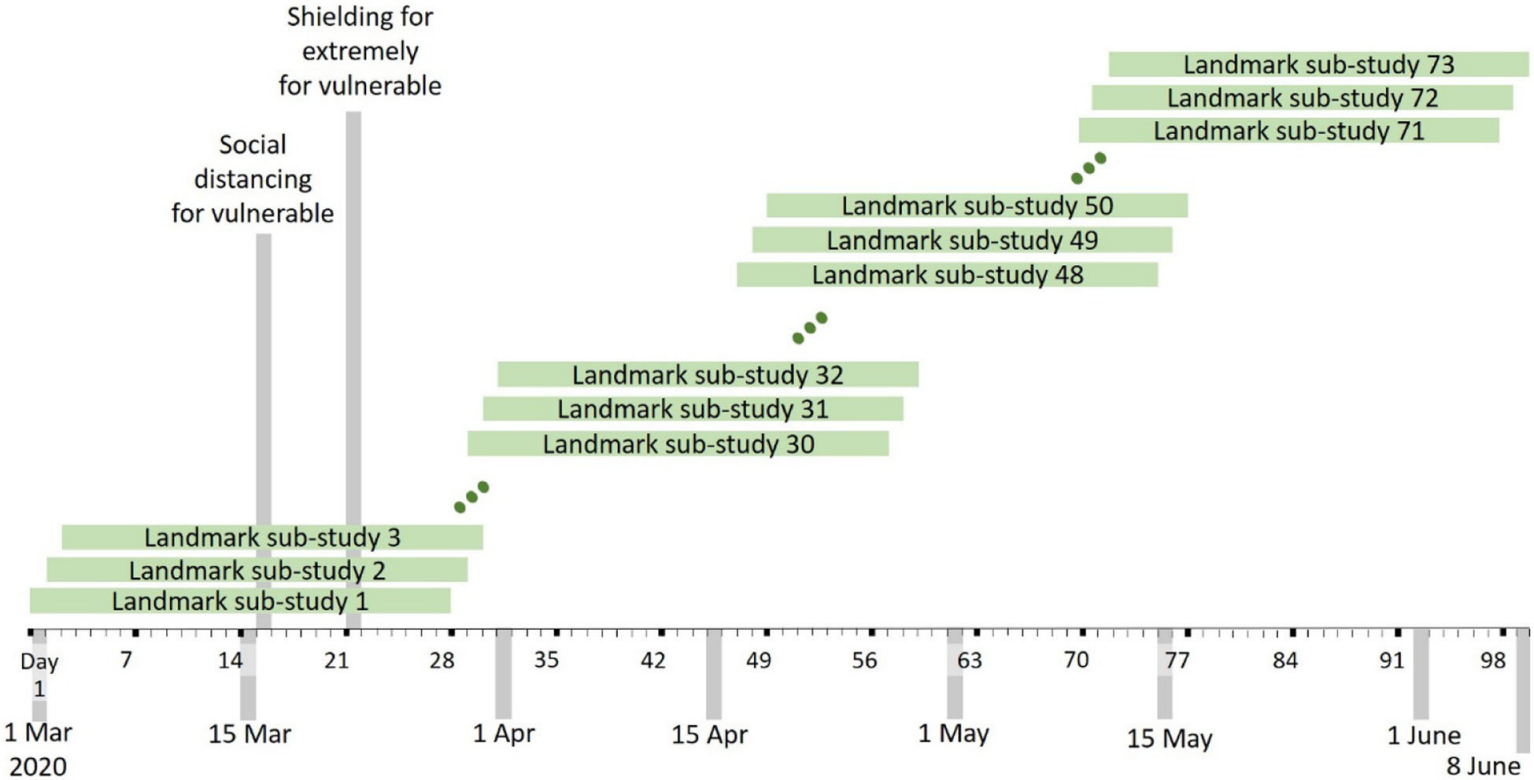
Schematic showing the design of the 28-day landmarking sub-studies (approach B)

To develop models within approach B, using landmarking models [7], a series of 73 overlapping sequential sub-studies were extracted from the base cohort. The sub-studies started 0, 1, 2, 3, 4…, 72 days after 1 March 2020 (Fig. 1). Follow-up started at the sub-study start date and ended at the first of COVID-19-related death or 28 days after sub-study entry. Individuals were not censored at deaths due to other causes. The outcome was COVID-19-related death during the 28-day period. Each sub-study had a case-cohort design, including all eligible individuals (those in the base cohort still alive at the sub-study start) who experienced a COVID-19-related death during the sub-study period and an age-stratified random sample of sub-study eligible individuals, with age group-specific sampling fractions equal to 1/70 of the sampling fractions for approach A. Data from all sub-studies were combined for analysis. Predictor variables were assessed at day 0 of each sub-study.

Functional forms for the proxy measures of COVID-19 infection prevalence were selected using Akaike’s Information Criterion, with our selection of candidate functional forms guided by our proposed model for COVID-19 death (Supplementary Appendix).

A Poisson model for 28-day COVID-19-related death was fitted to the combined dataset using Barlow weights and robust standard errors, incorporating predictors—chosen via the lasso for the “selected” model, pre-specified for the “age-sex”, “comorbidities” and “full” models—and proxy measures of COVID-19 infection prevalence. In each case, 3 models were fitted: one for each proxy measure.

The predicted risk is given by the same equation as for the Cox model, replacing the hazard ratios by incidence rate ratios and obtaining the baseline survivor function from the estimated constant coefficient.

### Model validation

Calibration and discrimination were assessed for 17 risk prediction algorithms (5 approach A; 12 approach B). For approach A, this was the “selected”, “age-sex”, “comorbidities” and “full” models, as well as the existing COVID-AGE risk tool (10th update) [13, 14]. For approach B, this was the “selected”, “age-sex”, “comorbidities” and “full” models incorporating each of the 3 proxy measures of infection prevalence.

### Overall internal validation

**Fig. 2:**
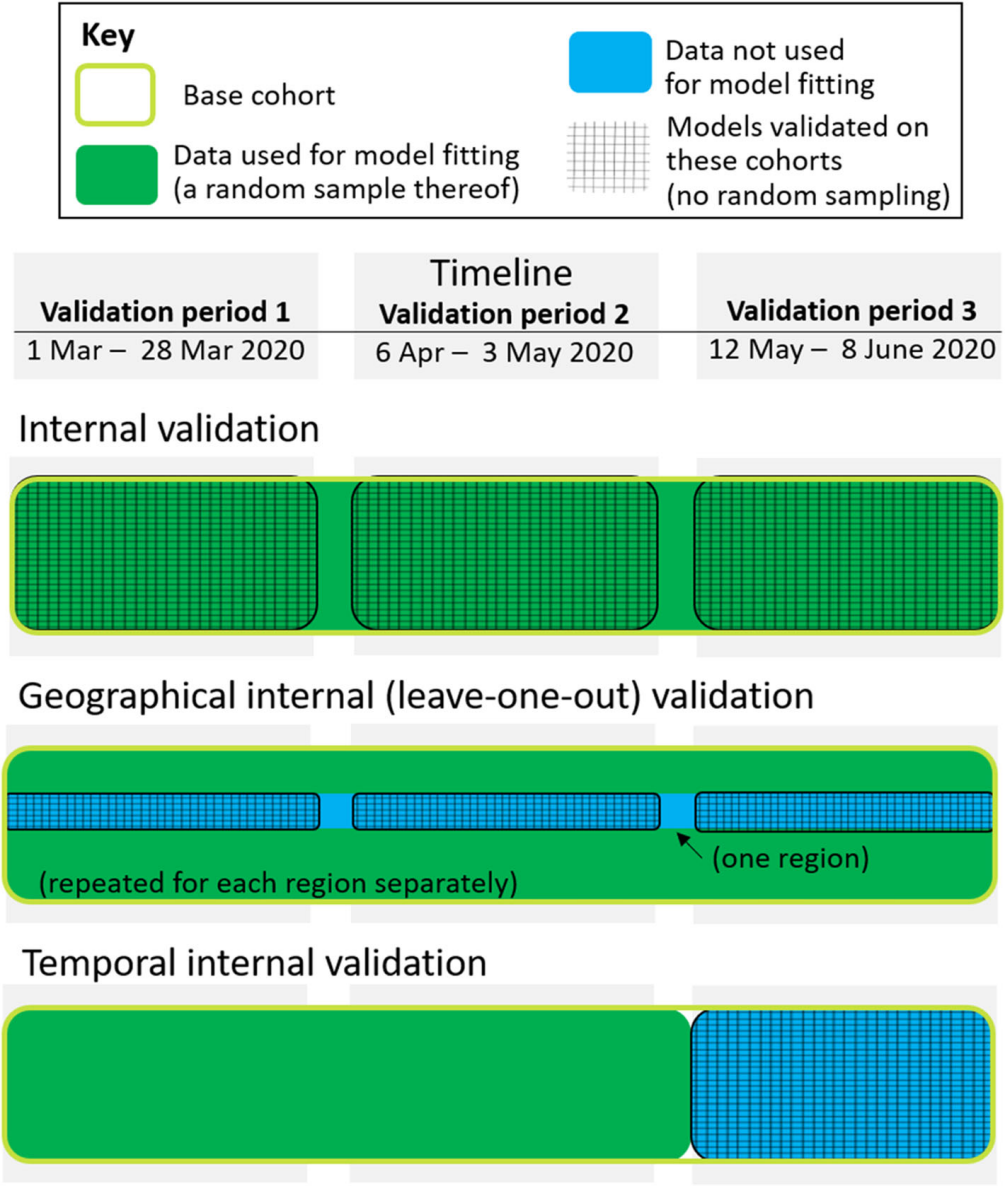
Schematic showing the forms of validation undertaken

From the base cohort, three validation cohorts were defined each lasting 28 days. The three validation cohorts covered periods with higher and lower infection prevalence (Fig. 2), to allow comparison of modelling strategies under these different conditions. Each validation cohort study comprised all individuals from the base cohort who remained alive at the start of the validation period. Predictor variables were assessed at day 0 of the validation cohort; predicted risk of 28-day COVID-19-related death was obtained using each of the 17 algorithms. Model performance was assessed by comparing these predicted risks to the observed binary outcome, 28-day COVID-19-related death (death within the 28-day period of the validation cohort).

Discrimination was assessed by Harrell’s C-statistic [15, 16]. Calibration was assessed by estimating the calibration intercept and slope and comparing observed and predicted risk, overall and by groups of predicted risk [16, 17]. Flexible calibration curves were drawn. Model performance was assessed overall and within sex and broad age groups (18–<70, 70–<80 and 80+; insufficient events occurred in the youngest age group to split further) and regions.

COVID-AGE is primarily a risk stratification tool, so we did not obtain calibration measures for this tool [14].

### Geographical and temporal internal validation

Although we were unable to perform external validation, we undertook additional forms of internal validation as recommended by Steyerberg et al. [18]. To assess model performance in different geographical contexts, the 16 risk prediction models developed within these data were re-fitted after excluding all individuals from a particular geographical region [18]. The resulting models were used to predict risk in the subset of the 3 validation cohorts comprising individuals from the excluded geographical region. Model performance was assessed within the excluded region (i.e. validation was performed using individuals whose data was not used in developing the risk prediction models). This process was repeated for each of the 7 regions.

To assess model performance in different temporal contexts, the 16 risk prediction models were re-fitted after excluding the last 28 days of data (i.e. the whole time period of validation period 3). The resulting models were used to predict risk in validation cohort 3. Individuals who remained alive at the start of validation cohort 3 appeared in both the model development data and the validation data, but their predictor values were updated at the start of validation period 3; individuals who experienced the event in the development data did not appear in the validation data.

### Missing data

Ethnicity, body mass index (BMI) and smoking data are collected in response to clinical need, thus likely to be missing not at random. As smoking and obesity, if present, are likely to be recorded, individuals with missing BMI were assumed non-obese and individuals with no smoking information were assumed non-smokers. Individuals with no serum creatinine measurement were included in the “no evidence of poor kidney function” group. Individuals with diabetes but no glycosylated haemoglobin (HbA1c) measurement were included in a separate “diabetes, no HbA1c” category. Analysis was restricted to individuals with recorded age, sex and ethnicity data; such complete case analysis is valid under a range of missing not at random assumptions [19].

### Sensitivity analysis

Additional sensitivity analyses were undertaken (Supplementary Appendix), including refitting the approach A “selected” model in the entire cohort (without case-cohort sampling), leading to very similar results, and using a Weibull model for both approaches A and B to increase comparability between approaches, resulting in similar conclusions about the comparison between approaches. Further details of the methods used can be found in our pre-published protocol [20].

### Software

Data management was performed using Python and Google BigQuery, with analysis carried out using Stata 16.1/Python. All code used is openly shared online for review and re-use under MIT open license (https://github.com/opensafely/risk-prediction-research).

## Patient and public involvement

We invite any patient or member of the public to contact us regarding this study or the broader OpenSAFELY project through our website https://opensafely.org/.

## Results

The base cohort comprised almost 12 million individuals, of whom 7999 experienced a COVID-19-related death (Table 1, Fig. S1 in the Supplementary Appendix). For model development, the approach A case-cohort study included 7999 COVID-19-related deaths and a sub-cohort of 319,917. Approach B sub-studies included all 7999 COVID-19 deaths and 330,132 individuals. The fitted models are provided in the Supplementary Appendix.

**Table 1:** Description of base cohort

	*N* (%)	COVID-19-related deaths (row %)
Total	11,972,947 (100.0)	7999 (0.07)
*Age group*		
18–39	4,275,852 (35.7)	52 (0.00)
40–49	2,022,527 (16.9)	141 (0.01)
50–59	2,040,181 (17.0)	437 (0.02)
60–69	1,635,143 (13.7)	938 (0.06)
70–79	1,319,367 (11.0)	2025 (0.15)
80+	679,877 (5.7)	4406 (0.65)
*Sex*		
Female	6,232,725 (52.1)	3315 (0.05)
Male	5,740,222 (47.9)	4684 (0.08)
*BMI*		
Underweight (<18.5)	249,294 (2.1)	306 (0.12)
Normal (18.5–29.9)	3,982,133 (33.3)	2480 (0.06)
Obese I (30–34.9)	3,690,583 (30.8)	2430 (0.07)
Obese II (35–39.9)	1,794,812 (15.0)	1462 (0.08)
Obese III (40+)	678,109 (5.7)	658 (0.10)
Missing^a^	1,242,341 (10.4)	340 (0.03)
*Smoking*		
Never smoker	5,540,732 (46.3)	2499 (0.05)
Former smoker	3,921,016 (32.7)	4745 (0.12)
Current smoker	2,253,231 (18.8)	737 (0.03)
Missing	257,968 (2.2)	18 (0.01)
*Ethnicity*		
White	10,184,871 (85.1)	6952 (0.07)
Indian	405,477 (3.4)	299 (0.07)
Pakistani	262,882 (2.2)	161 (0.06)
Bangladeshi/other Asian	262,882 (2.2)	161 (0.06)
African/other black	280,466 (2.3)	173 (0.06)
Caribbean	80,863 (0.7)	124 (0.15)
Chinese	103,423 (0.9)	20 (0.02)
Mixed/others	392,097 (3.3)	147 (0.04)
*Deprivation*		
IMD 1 (least deprived)	2,315,449 (19.3)	1255 (0.05)
IMD 2	2,375,974 (19.8)	1443 (0.06)
IMD 3	2,398,815 (20.0)	1537 (0.06)
IMD 4	2,489,997 (20.8)	1791 (0.07)
IMD 5 (most deprived)	2,392,712 (20.0)	1973 (0.08)
*Location*		
Urban	9,595,617 (80.1)	6775 (0.07)
Rural	2,377,330 (19.9)	1224 (0.05)
*Region*		
East	2,730,203 (22.8)	1773 (0.06)
London	1,018,332 (8.5)	755 (0.07)
Midlands	2,673,963 (22.3)	2005 (0.07)
North East and Yorkshire	2,242,375 (18.7)	1679 (0.07)
North West	1,053,537 (8.8)	905 (0.09)
South East	744,930 (6.2)	309 (0.04)
South West	1,509,607 (12.6)	573 (0.04)
*Blood pressure*		
Normal	2,797,632 (23.4)	1725 (0.06)
Elevated	1,757,455 (14.7)	1406 (0.08)
High, stage I	3,899,203 (32.6)	2454 (0.06)
High, stage II	2,492,161 (20.8)	2389 (0.10)
Missing	1,026,496 (8.6)	25 (0.00)
Diagnosed hypertension	2,448,605 (20.5)	5332 (0.22)
**Comorbidities**		
***Respiratory***		
Asthma, no OCS use	1,829,710 (15.3)	1008 (0.06)
Asthma, with OCS use	112,407 (0.9)	248 (0.22)
Respiratory disease	495,699 (4.1)	1809 (0.36)
Cystic fibrosis or other conditions	3167 (0.0)	4 (0.13)
***Cardiovascular***		
Cardiac disease	783,896 (6.5)	3008 (0.38)
Atrial fibrillation	430,798 (3.6)	1808 (0.42)
DVT/PE	249,969 (2.1)	773 (0.31)
PAD or lower limb amputation	41,362 (0.3)	220 (0.53)
Diabetes, controlled	738,369 (6.2)	1908 (0.26)
Diabetes, uncontrolled	346,726 (2.9)	1054 (0.30)
Diabetes, status unknown	133,387 (1.1)	306 (0.23)
***Neurological***		
Stroke	240,401 (2.0)	1287 (0.54)
Vascular dementia	22,792 (0.2)	489 (2.15)
Other neurological conditions	114,431 (1.0)	480 (0.42)
***Cancer (non-haematological)***		
Diagnosed < 1 year ago	54,290 (0.5)	216 (0.40)
Diagnosed 2–5 years ago	157,859 (1.3)	358 (0.23)
Diagnosed 5+ years ago	362,457 (3.0)	879 (0.24)
***Haematological cancer***		
Diagnosed <1 year ago	6151 (0.1)	47 (0.76)
Diagnosed 2–5 years ago	18,722 (0.2)	95 (0.51)
Diagnosed 5+ years ago	42,492 (0.4)	116 (0.27)
***Kidney and liver***		
Reduced kidney function (eGFR in range 30–<60 mL/min/1.73m^2^)	607,308 (5.1)	2793 (0.46)
Very reduced kidney function (eGFR <30 mL/min/1.73m^2^)	58,081 (0.5)	694 (1.19)
Dialysis	8871 (0.1)	115 (1.30)
Liver disease	74,193 (0.6)	189 (0.25)
Organ transplant	11,349 (0.1)	50 (0.44)
***Immunosuppression***		
Spleen	19,815 (0.2)	29 (0.15)
RA/SLE/psoriasis	609,421 (5.1)	733 (0.12)
Immunosuppression	13,091 (0.1)	28 (0.21)
HIV	23,078 (0.2)	17 (0.07)
Inflammatory bowel disease	152,080 (1.3)	169 (0.11)
***Others***		
Fracture (in >65 year old in last 2 years)	55,952 (0.5)	443 (0.79)
Learning disability	158,350 (1.3)	161 (0.10)
Serious mental illness	150,928 (1.3)	254 (0.17)

*BMI *Body Mass Index, *IMD *Index of Multiple Deprivation, *OCS *oral corticosteroids, *eGFR *estimated glomerular filtration rate, *DVT/PE *Deep vein thrombosis/Pulmonary embolism, *PAD *Peripheral arterial disease, *RA/SLE *Rheumatoid arthritis/Systemic lupus erythematosus

^a^Combined with normal category into a “no evidence of overweight/underweight” for modelling

### Discrimination

In all validation periods, the C-statistic for the “selected” models was high (0.92–0.94), indicating excellent ability to distinguish between individuals who experienced 28-day COVID-19 death and who did not (Table 2). No difference in discrimination was seen between approaches A and B.

**Table 2:** Measures of model performance in predicting 28-day risk of COVID-19 mortality, using the “selected” models

Approach; measures of infection prevalence included; model form	Validation period	COVID-19 deaths/sample size	C-statistic	Observed mean risk (%)	Predicted mean risk (%)	Calibration	
						Intercept (95% *CI*)	Slope (95% *CI*)
(A), none, Cox	1	455/11,972,492	0.924	0.0038	0.0038	0.00 (−0.10, 0.09)	0.95 (0.86, 1.05)
	2	4471/11,955,296	0.934	0.0374	0.0038	2.30 (2.27, 2.32)	1.02 (0.99, 1.05)
	3	1246/11,942,608	0.941	0.0104	0.0037	1.03 (0.97, 1.08)	1.05 (1.00, 1.11)
(B), modelled estimates, Poisson	1	455/11,972,492	0.925	0.0038	0.0044	−0.15 (−0.24, −0.06)	0.93 (0.84, 1.02)
	2	4471/11,955,296	0.937	0.0374	0.0354	0.06 (0.03, 0.09)	1.00 (0.97, 1.03)
	3	1246/11,942,608	0.944	0.0104	0.0128	−0.20 (−0.26, −0.15)	1.03 (0.98, 1.09)
(B), A&E COVID-19 attendances, Poisson	1	455/11,972,492	0.921	0.0038	0.0145	−1.34 (−1.43, −1.25)	0.92 (0.83, 1.02)
	2	4471/11,955,296	0.933	0.0374	0.0420	−0.12 (−0.15, −0.09)	0.99 (0.96, 1.02)
	3	1246/11,942,608	0.943	0.0104	0.0197	−0.64 (−0.69, −0.58)	1.05 (0.99, 1.10)
(B), Suspected COVID-19 in primary care, Poisson	1	455/11,972,492	0.921	0.0038	0.0085	−0.80 (−0.89, −0.71)	0.90 (0.81, 1.00)
	2	4471/11,955,296	0.935	0.0374	0.0378	−0.01 (−0.04, 0.02)	1.00 (0.97, 1.03)
	3	1246/11,942,608	0.942	0.0104	0.0156	−0.41 (−0.46, −0.35)	1.04 (0.98, 1.09)

Among the oldest age group (80+ years), discrimination was much lower than in younger age groups (Table 3), reflecting the substantial discrimination that comes from age which is partly lost after age restriction. For example, for the approach A “selected” model in validation period 1, the C-statistic was 0.77 for females and 0.65 for males among the 80+ age group compared with 0.88 and 0.91 among the 18–<70 age group. Discrimination was similar across regions (Table S4; range of C-statistics across regions and validation periods: approach A 0.903–0.962, approach B 0.907–0.966, other than in the North West region in validation period 1, which had lower discrimination: approach A C-statistic 0.84; approach B 0.85–0.86).

**Table 3:** C-statistics for different sets of predictor variable sets, by sex and broad age group

Approach; measures of infection prevalence included; model form	Validation period	Predictor set	C-statistic					
			Age 18–<70		Age 70–<80		Age 80+	
			Female	Male	Female	Male	Female	Male
(A), none, Cox	1	Age-sex	0.809	0.834	0.580	0.575	0.641	0.533
		Comorbidities	0.887	0.917	0.842	0.772	0.772	0.641
		COVID-AGE	0.908	0.915	0.833	0.762	0.720	0.631
		Selected	0.876	0.910	0.838	0.785	0.765	0.651
		Full	0.892	0.929	0.846	0.789	0.762	0.662
	2	Age-sex	0.794	0.815	0.593	0.560	0.631	0.633
		Comorbidities	0.895	0.888	0.775	0.730	0.705	0.688
		COVID-AGE	0.906	0.888	0.765	0.733	0.698	0.679
		Selected	0.888	0.892	0.813	0.776	0.746	0.731
		Full	0.921	0.915	0.835	0.796	0.776	0.763
	3	Age-sex	0.832	0.819	0.570	0.600	0.655	0.650
		Comorbidities	0.905	0.890	0.784	0.735	0.706	0.708
		COVID-AGE	0.907	0.897	0.808	0.721	0.711	0.701
		Selected	0.910	0.890	0.837	0.792	0.747	0.750
		Full	0.918	0.908	0.856	0.830	0.780	0.783
(B), modelled estimates, Poisson	1	Age-sex	0.814	0.851	0.709	0.634	0.723	0.607
		Comorbidities	0.884	0.918	0.856	0.773	0.790	0.668
		Selected	0.862	0.906	0.861	0.765	0.789	0.679
		Full	0.890	0.928	0.863	0.786	0.786	0.688
	2	Age-sex	0.808	0.828	0.625	0.583	0.634	0.648
		Comorbidities	0.897	0.889	0.778	0.733	0.706	0.697
		Selected	0.894	0.895	0.825	0.786	0.767	0.751
		Full	0.923	0.915	0.836	0.799	0.777	0.765
	3	Age-sex	0.835	0.836	0.638	0.611	0.692	0.684
		Comorbidities	0.900	0.897	0.797	0.740	0.724	0.727
		Selected	0.900	0.889	0.852	0.794	0.781	0.772
		Full	0.919	0.908	0.863	0.824	0.793	0.792

Geographical internal validation showed that discrimination of models was largely insensitive to the removal of a region (Tables 4 and S7, Figure S9), with the exception of omission of the North-West region in validation period 1, which resulted in lower discrimination for all models. Removing validation period 3 from the development data, in the temporal validation, did not reduce discrimination for validation period 3.

**Table 4:** Measures of model performance in predicting 28-day risk of COVID-19 mortality in the temporal and geographical internal validation, using the “selected” models

Approach; measures of infection prevalence included; model format	Validation period	Region omitted from analysis	C-statistic	Observed mean risk (in validation data) (%)	Predicted mean risk (in validation data) (%)	Calibration	
						Intercept (95% *CI*)	Slope (95% *CI*)
			**Temporal internal validation**				
(A), none, Cox	3	–	0.941	0.0104	0.0037	1.03 (0.97, 1.08)	1.05 (1.00, 1.11)
(B), modelled estimates, Poisson	3	–	0.943	0.0104	0.0195	−0.63 (−0.68, −0.57)	1.04 (0.98, 1.09)
			**Geographical internal validation**				
(A), none, Cox	1	East	0.907	0.0036	0.0034	0.05 (−0.15, 0.25)	0.85 (0.65, 1.05)
		London	0.948	0.0102	0.0024	1.46 (1.27, 1.65)	0.98 (0.78, 1.17)
		Midlands	0.927	0.0052	0.0038	0.31 (0.14, 0.48)	0.95 (0.78, 1.12)
		North East and Yorkshire	0.896	0.0024	0.0046	−0.66 (−0.92, −0.39)	0.81 (0.55, 1.08)
		North West	0.857	0.0021	0.0045	−0.78 (−1.19, −0.36)	0.72 (0.31, 1.14)
		South East	0.946	0.0025	0.0035	−0.33 (−0.78, 0.12)	1.06 (0.61, 1.51)
		South West	0.933	0.0014	0.0042	−1.10 (−1.53, −0.67)	0.92 (0.50, 1.35)
	2	East	0.930	0.0365	0.0034	2.39 (2.33, 2.45)	1.05 (0.99, 1.11)
		London	0.932	0.0396	0.0023	2.84 (2.74, 2.94)	0.90 (0.80, 1.00)
		Midlands	0.931	0.0405	0.0038	2.39 (2.33, 2.45)	0.99 (0.93, 1.05)
		North East and Yorkshire	0.937	0.0420	0.0046	2.22 (2.16, 2.28)	1.00 (0.94, 1.07)
		North West	0.931	0.0493	0.0045	2.40 (2.32, 2.49)	1.02 (0.94, 1.11)
		South East	0.938	0.0238	0.0035	1.92 (1.77, 2.07)	1.04 (0.89, 1.19)
		South West	0.941	0.0235	0.0041	1.74 (1.64, 1.84)	1.05 (0.94, 1.15)
	3	East	0.942	0.0106	0.0033	1.17 (1.05, 1.28)	1.08 (0.97, 1.20)
		London	0.947	0.0033	0.0023	0.37 (0.04, 0.71)	0.91 (0.57, 1.24)
		Midlands	0.935	0.0120	0.0037	1.18 (1.07, 1.29)	0.99 (0.88, 1.10)
		North East and Yorkshire	0.945	0.0142	0.0045	1.15 (1.04, 1.26)	1.04 (0.93, 1.15)
		North West	0.935	0.0168	0.0044	1.33 (1.19, 1.48)	1.06 (0.92, 1.21)
		South East	0.965	0.0052	0.0035	0.42 (0.10, 0.73)	1.20 (0.89, 1.52)
		South West	0.920	0.0047	0.0041	0.15 (−0.09, 0.38)	1.07 (0.83, 1.30)
(B), modelled estimates, Poisson	1	East	0.913	0.0036	0.0020	0.57 (0.37, 0.77)	0.87 (0.68, 1.07)
		London	0.952	0.0102	0.0081	0.23 (0.04, 0.42)	0.99 (0.80, 1.19)
		Midlands	0.924	0.0052	0.0058	−0.12 (−0.29, 0.04)	0.91 (0.75, 1.08)
		North East and Yorkshire	0.907	0.0024	0.0035	−0.37 (−0.64, −0.10)	0.86 (0.59, 1.12)
		North West	0.857	0.0021	0.0045	−0.77 (−1.19, −0.36)	0.71 (0.30, 1.13)
		South East	0.937	0.0025	0.0048	−0.63 (−1.08, −0.18)	1.01 (0.56, 1.45)
		South West	0.931	0.0014	0.0047	−1.21 (−1.64, −0.79)	0.93 (0.50, 1.35)
	2	East	0.936	0.0365	0.0356	0.03 (−0.04, 0.09)	1.02 (0.96, 1.09)
		London	0.937	0.0396	0.0429	−0.08 (−0.18, 0.02)	0.93 (0.83, 1.02)
		Midlands	0.933	0.0405	0.0373	0.08 (0.02, 0.14)	0.99 (0.93, 1.05)
		North East and Yorkshire	0.939	0.0420	0.0352	0.18 (0.11, 0.24)	0.99 (0.93, 1.06)
		North West	0.931	0.0493	0.0392	0.23 (0.15, 0.32)	0.99 (0.91, 1.08)
		South East	0.943	0.0238	0.0319	−0.30 (−0.44, −0.15)	1.04 (0.89, 1.19)
		South West	0.941	0.0235	0.0277	−0.17 (−0.27, −0.06)	1.02 (0.91, 1.12)
	3	East	0.943	0.0106	0.0103	0.03 (−0.09, 0.14)	1.05 (0.93, 1.17)
		London	0.954	0.0033	0.0102	−1.12 (−1.46, −0.78)	0.94 (0.60, 1.28)
		Midlands	0.938	0.0120	0.0153	−0.24 (−0.35, −0.13)	0.99 (0.88, 1.10)
		North East and Yorkshire	0.944	0.0142	0.0140	0.01 (−0.10, 0.12)	1.03 (0.92, 1.14)
		North West	0.935	0.0168	0.0205	−0.20 (−0.35, −0.05)	1.03 (0.88, 1.18)
		South East	0.965	0.0052	0.0110	−0.74 (−1.05, −0.42)	1.16 (0.85, 1.48)
		South West	0.921	0.0047	0.0071	−0.41 (−0.64, −0.18)	1.04 (0.81, 1.27)

### Absolute risk estimation

For the approach A Cox model, the mean predicted and observed risks were very similar in the first validation period (the initial portion of the data on which the model was fitted), but different in the second and third (Table 2). In validation period 2, the mean observed risk was ten times higher than in validation period 1, but the mean predicted risk was (by design, since it ignores the infection prevalence) almost identical for all three periods.

For the approach B models incorporating model-based estimates of infection prevalence, the mean and observed risks were very similar in all validation periods. The calibration intercept was slightly less than zero in validation periods 1 and 3, indicating slight over-estimation on average, with calibration slopes close to one. Replacing model-based estimates by either the rate of A&E COVID-19 attendances or the rate of suspected COVID-19 cases in primary care resulted in poorer calibration, particularly in the first validation period which had a very low infection prevalence. All approach B models had worse calibration than the approach A model in validation period 1 but considerably better calibration than the approach A model in the other two validation periods. Figures S3-S6 show flexible calibration curves and predicted versus observed risks by twentieths of predicted risk.

Calibration intercepts varied by region (e.g. approach A in validation period 1, range of estimated calibration intercepts: −1, +1.12), but calibration slopes varied less (Table S4). The intercepts varied least for approach B in validation period 2 (range of estimated calibration intercepts: −0.28, 0.21), which was the period with the highest infection prevalence.

Geographical internal validation showed that estimated calibration intercepts were sensitive to removal of a geographical region, but slopes less so. Using approach A models, the temporal validation results were similar to the overall internal validation findings. In contrast, using approach B, we found worse calibration under the temporal validation (Table S6, Fig. S8).

### Models with fewer predictors

A simple model including only age and sex provided reasonable discrimination (C-statistic ~0.80) among the 18–<70 age group (Tables 3 and S5). Among older age groups, in contrast, discrimination was substantially lower in the “age-sex” model compared to the models with more predictors.

Among females in the 18–<70 age group, both the “comorbidities” and “full” models had C-statistics of 0.89 in validation period 1. In males, the analogous numbers were 0.92 (“comorbidities”) and 0.93 (“full”). Among this age group, across the validation periods in both approaches A and B, discrimination was at most 0.03 lower for the “comorbidities” compared to the “full” model.

The COVID-AGE algorithm had C-statistics at most 0.03 lower than the most complex models used among the 18–<70 age group (e.g. validation period 1 C-statistic 0.91 (“COVID-AGE”) vs 0.89 (“full”) among females; 0.92 (“COVID-AGE”) vs 0.93 (“full”) among males). In older age groups, the “full” model had higher discrimination than “COVID-AGE” (e.g. validation period 2 C-statistic 0.70 (“COVID-AGE”) vs 0.78 (“full”) among females; 0.68 (“COVID-AGE”) vs 0.76 (“full”) among males).

## Discussion

We have proposed a modelling approach based on landmarking which enables dynamic incorporation of time- and region-dependent information on infection prevalence. We have demonstrated that our modelling approach can provide well-calibrated estimates, with good discrimination, of absolute risk of 28-day COVID-19 death in the general population. In contrast, absolute risk estimates cannot be transported from models which do not explicitly model the infection prevalence, although they did rank individuals well. We demonstrated that the performance of our proposed modelling approach depended critically on the proxy used for infection prevalence. We found that the calibration of our modelling approach worsened when applied to time periods with patterns of infection not seen in the model development data. We did not undertake external validation; performance may worsen in completely new settings.

Identifying a readily available proxy of infection prevalence is challenging in practice. Models including model-based estimates provided the best performance, but this may be impractical for most situations due to the complex modelling required to obtain these estimates. Models using proxies more readily available through automated data streams gave slightly inferior performance, albeit considerably better than ignoring the burden of infection entirely. Proxies involving more serious consequences of infection, such as A&E attendance, performed poorly when the underlying infection prevalence was low, since these serious consequences are rare and therefore imprecisely estimated in low prevalence periods. Furthermore, one limitation of this work was that we were unable to explore the level of granularity required in the data used as a proxy for infection prevalence. Where COVID-19 testing is geographically widespread, direct estimates of local infection are likely to provide the best measure. While this was unavailable in the UK at our study start, more recent testing data are much more comprehensive.

Joint modelling offers an alternative approach to obtaining dynamic predictions [21]. We chose a landmarking approach because it is computationally efficient and it is easier to extend to make predictions beyond the temporal subset of data used for model development.

Our underlying theoretical model for COVID-19-related death suggested it may be possible to separate the dynamics of the epidemic from the process of risk prediction based on patient characteristics, allowing the infection process to be ignored, provided that models carefully account for geographical region. Surprisingly, we found that our approach A models, which did not account for region, were still able to provide good discrimination which transported well geographically and temporally. This provides reassurance that these potential theoretical biases do not lead to substantial deterioration in practical performance in terms of ranking patients, but region-specific performance suggested accounting for region may be important when good calibration is desired.

We found that the existing COVID-AGE tool [13, 14] had very high discrimination, similar to the best performing models considered, suggesting this provides a reliable ranking of COVID-19 mortality risk. Interestingly, very simple models including only age, sex, ethnicity, a rural indicator, and a count of total comorbidities led to models with very good discrimination. When focusing on specific patient groups with higher morbidity, more complex models may provide useful additional discrimination, but in many cases much simpler models are able to discriminate well.

We did not correct any of the models for optimism [22, 23], although the similarity of the C-statistics in the overall and geographical internal validations suggests optimism was not a major problem. We compared a number of models that differed in terms of parsimony, ranging from no variable selection to a model containing only age and sex. The regression penalization used in the selected model has the effect of shrinking model coefficients; however, over-optimism can still remain [24].

Electronic health record data are not collected for research, so information on certain characteristics can be incomplete or absent. Our approach to missing data reflected the way in which these models might be used in practice if applied within electronic health record systems. Our measures of model performance reflect performance under this implementation [25]. Differences observed between approaches are unlikely to have been affected by the approach taken to missing data.

## Conclusion

The pandemic and our response to it are evolving rapidly. As vaccines and booster programmes roll out, risk prediction models will need to be updated and re-calibrated. Our results have a number of implications for researchers developing risk prediction algorithms in COVID-19. First, models that do not explicitly model the infection prevalence can provide good discrimination which transports well geographically and temporally. However, the calibration of such models is poor when applied to different temporal and geographical contexts. When calibration is important, models must explicitly model the infection prevalence. However, performance of these models is very sensitive to the quality of the proxy measure used. Furthermore, our temporal validation suggested that for these models, calibration declines when applied to data which has patterns of infection prevalence not seen in the data used to develop the model, such as occurred in our third validation period. Discrimination, conversely, did not decline. For all models, region-specific calibration was worse than overall calibration, suggesting that there may be regional differences which are important for COVID-19 risk prediction beyond (our measures of) infection levels. While we have focused on COVID-19 death, these findings are relevant for models predicting other severe outcomes of COVID-19 in the general population, such as hospitalisation.

Finally, most of the discriminating power in each model evaluated here was driven by simple features such as age, sex and a count of comorbidities. Complex risk prediction models driven by multiple variables from diverse sources can be difficult, slow and expensive to implement in routine care: our results suggest that the opportunity costs and complexity of such implementations may not be warranted. The finding that simple models produce very high discrimination also suggests that policies targeting population-level reduction of COVID-19 mortality risk may not need to distinguish between all comorbidities in detail. For policy decisions, including ongoing restrictions on social contact and return-to-work strategies, a simple approach to predicting risk incorporating simple eligibility criteria, could accelerate programme rollout and delivery of policies.

## Supplementary Information

**Additional file 1.** Supplementary materials

## Data Availability

Access to the underlying identifiable and potentially re-identifiable pseudonymised electronic health record data is tightly governed by various legislative and regulatory frameworks and restricted by best practice. The data in OpenSAFELY is drawn from General Practice data across England where TPP is the Data Processor. TPP developers (CB, RC, JP, FH, and SH) initiate an automated process to create pseudonymised records in the core OpenSAFELY database, which are copies of key structured data tables in the identifiable records. These are linked onto key external data resources that have also been pseudonymised via SHA-512 one-way hashing of NHS numbers using a shared salt. DataLab developers and PIs (BG, LS, CEM, SB, AJW, WJH, DE, PI, and CTR) holding contracts with NHS England have access to the OpenSAFELY pseudonymised data tables as needed to develop the OpenSAFELY tools. These tools in turn enable researchers with OpenSAFELY Data Access Agreements to write and execute code for data management and data analysis without direct access to the underlying raw pseudonymised patient data and to review the outputs of this code. All code for the full data management pipeline—from raw data to completed results for this analysis—and for the OpenSAFELY platform as a whole is available for review at github.com/OpenSAFELY.

## Abbreviations

A&E: Accident and Emergency Department
GP: General practitioner
ICD: International Classification of Diseases
NHS: National Health Service (UK)
ONS: Office for National Statistics
