# Supplementary material for: Comparison of methods for predicting COVID-19-related death in the general population using the OpenSAFELY platform
Source: Diagn Progn Res. 2022 Feb 24;6:6. doi: 10.1186/s41512-022-00120-2 (PMC8865947; doi:10.1186/s41512-022-00120-2)
Supplement: Supplementary file 1 — Additional file 1. Supplementary materials [file 41512_2022_120_MOESM1_ESM.docx]

Comparison of methods for predicting COVID-19 related death using the OpenSAFELY platform

SUPPLEMENTARY MATERIALS

3^rd^ February 2022

The OpenSAFELY Collaborative, Elizabeth J Williamson^1^**, John Tazare^1^**, Krishnan Bhaskaran^1^, Helen I McDonald^1,2^, Alex J Walker^3^, Laurie Tomlinson^1^, Kevin Wing^1^, Sebastian Bacon^3^, Chris Bates^4^, Helen J Curtis^3^, Harriet J Forbes^1^, Caroline Minassian^1^, Caroline E Morton^3^, Emily Nightingale^1^, Amir Mehrkar^3^, David Evans^3^, Brian D Nicholson^3^, David A Leon^1^, Peter Inglesby^3^, Brian MacKenna^3^, Nicholas G Davies^1^, Nicholas J DeVito^3^, Henry Drysdale^3^, Jonathan Cockburn^4^, William J Hulme^3^, Jess Morley^3^, Ian Douglas^1^, Christopher T Rentsch^1^, Rohini Mathur^1^, Angel Wong^1^, Anna Schultze^1^, Richard Croker^3^, John Parry^4^, Frank Hester^4^, Sam Harper^4^, Richard Grieve^1^, David A Harrison^5^, Ewout W. Steyerberg^6^, Rosalind M Eggo^1^, Karla Diaz-Ordaz^1^, Ruth Keogh^1^, Stephen JW Evans^1^, Liam Smeeth^1^†, Ben Goldacre^3^† (ORCID: 0000-0002-5127-4728).

† Joint principal investigators; ** joint first authors.

1. London School of Hygiene and Tropical Medicine, Faculty of Epidemiology & Population Health, Keppel Street, London WC1E 7HT

2. NIHR Health Protection Research Unit (HPRU) in Immunisation

3. The DataLab, Nuffield Department of Primary Care Health Sciences, University of Oxford, OX26GG

4. TPP, TPP House, 129 Low Lane, Horsforth, Leeds, LS18 5PX

5. Intensive Care National Audit & Research Centre (ICNARC), 24 High Holborn, Holborn, London WC1V 6AZ

6. Leiden University Medical Center, Leiden, the Netherlands

Correspondence to:

Elizabeth Williamson

[Elizabeth.williamson@lshtm.ac.uk](mailto:Elizabeth.williamson@lshtm.ac.uk)

London School of Hygiene and Tropical Medicine, Faculty of Epidemiology & Population Health, Keppel Street, London WC1E 7HT

Key words

Risk prediction; risk stratification; mortality; COVID-19; infectious disease; statistical methodology.

Contents

[1 Model for rate of COVID-19 over time 4](#_Toc89184452)

[1.1 Rate of infectious contacts 4](#_Toc89184453)

[1.2 Rate of infection 4](#_Toc89184454)

[1.3 Rate of COVID-19 related death 4](#_Toc89184455)

[1.4 Overall rate of death from COVID-19 5](#_Toc89184456)

[1.5 Separating the infection process from the clinical risk prediction 5](#_Toc89184457)

[1.6 Explicitly incorporating proxy measures of the burden of infection 5](#_Toc89184458)

[2 Potential predictors 7](#_Toc89184459)

[2.1 Demographic and clinical predictors: additional details 7](#_Toc89184460)

[2.2 Proxy measures of the prevalence of COVID-19 infection: additional details 8](#_Toc89184461)

[2.3 Predictors used in the simpler models 8](#_Toc89184462)

[2.4 Limitations in predictors for COVID-AGE 9](#_Toc89184463)

[2.5 Details of selection of predictors 9](#_Toc89184464)

[3 Additional sensitivity analyses undertake 13](#_Toc89184465)

[3.1 Comparing the cohort and case-cohort design 13](#_Toc89184466)

[3.2 Additional model types 13](#_Toc89184467)

[3.3 Combining proxy measures of the burden of COVID-19 infection 13](#_Toc89184468)

[3.4 Not updating patient characteristics in landmark sub-studies 13](#_Toc89184469)

[3.5 Removing the proxies of burden of infection 13](#_Toc89184470)

[4 Study flowchart 14](#_Toc89184471)

[5 Estimated regression coefficients 15](#_Toc89184472)

[6 Model performance: internal validation 19](#_Toc89184473)

[7 Geographical and temporal internal validation results 30](#_Toc89184474)

[8 Protocol deviations 36](#_Toc89184475)

[9 Additional sensitivity analysis results 36](#_Toc89184476)

[9.1 Comparing the cohort and case-cohort design 36](#_Toc89184477)

[9.2 Additional model types 36](#_Toc89184478)

[9.3 Combining proxy measures of the burden of COVID-19 infection 37](#_Toc89184479)

[9.4 Not updating patient characteristics in landmark sub-studies 37](#_Toc89184480)

[9.5 Removing the proxies of burden of infection 37](#_Toc89184481)

[10 Third approach: daily landmarking 42](#_Toc89184482)

[10.1 Design 42](#_Toc89184483)

[10.2 Statistical analysis 42](#_Toc89184484)

[10.3 Results 42](#_Toc89184485)

[11 References 45](#_Toc89184486)

# Model for rate of COVID-19 over time

Let x be a vector containing the patient level predictors of interest (e.g. demographic information, comorbidities, medications, etc.), containing a constant term. The phrase “patient groups” is used below to mean that strata that are obtained by looking at individuals with particular values of the covariates x. Time (t) is measured as calendar time, t>0, representing the number of days from a pre-specified calendar date nominated to act as the “start” of the epidemic.

Suppose that the journey from becoming infected to dying with COVID-19 takes a maximum of M days and a minimum of m days. For example, m=0, M=14 represents the assumption that COVID-19 infection could cause death from the day of infection until two weeks after.

For an individual, the rate of death from COVID-19 on day t can be written:

$$\lambda\left( t \right)=\sum_{i=m}^{M} \lambda_{C}\left( t-i \right) \lambda_{I|C} \lambda_{D|I}(i)$$

- $\lambda_{C}\left( j \right)$ is the rate of being in contact with an infected person on day j.
- $\lambda_{I|C}$ is the rate of infection given a contact with an infected person.
- $\lambda_{D|I}(j)$ is the hazard of dying today given infection occurred j days previously.

## Rate of infectious contacts

Suppose the following is a reasonable model for the rate of contact:

$$\lambda_{C}\left( j \right)=e^{w^{'}x}f(j;x)$$

where

- $e^{w^{'}x}$ models the relative rate of contacts experienced by different patient groups (e.g. older age groups may, on average, experience fewer daily contacts than younger groups). This model assumes these relative rates remain constant over time.
- $f(j;x)$ is the proportion of the individual’s potential contacts who are infected on day j. This may depend on certain patient characteristics (e.g. region the patient lives in).

## Rate of infection

To become infected, given an infectious contact, the individual must be “susceptible” to infection and must then contract the infection. The probability of the former is likely to vary by patient group. The probability of the latter (contracting infection given susceptible) we assume constant over time. Therefore, our model is:

$$\lambda_{I| C}\left( j \right)= e^{\beta^{'}x} u\left( j;x \right)$$

where

- $u\left( j;x \right)$ is the probability that an individual in patient group x is susceptible on day j
- $e^{\beta^{'}x}$ captures the relative susceptibility to infection given contact across patient groups

## Rate of COVID-19 related death

We assume that the rate of COVID-19 death depends only on the number of days since infection. This is a simplification, since the hazard of death could additionally depend on the current day due to – for example – current lack of availability of ventilators.

$$\lambda_{D|I}\left( i \right)=\eta_{i} e^{\gamma^{'}x}$$

where

- $\eta_{i}$is the baseline rate of dying given infection occurred i days earlier.
- $e^{\gamma^{'}x}$ captures relative differences in the rate of death among different patient groups. These relative differences are assumed to be the same, no matter how long ago infection occurred.

## Overall rate of death from COVID-19

Substituting these sub-models into the overall rate of death from COVID-19, we get

$$\lambda\left( t \right)=e^{\left( w+\beta+\gamma\right)^{'}x} \sum_{i=m}^{M} u\left( t-i;x \right)f\left( t-i;x \right) \eta_{i}$$

We collect together all time-fixed terms related to patient groups:

$$\alpha=w+\beta+\gamma$$

And write

$$g\left( t;x,m,M \right)= \sum_{i=m}^{M} f^{*}\left( t-i;x \right)\eta_{i} where f^{*}\left( j;x \right)=u\left( j;x \right)f\left( j;x \right)$$

Our model for the rate of COVID-19 death at t days is:

|  | $\lambda\left( t \right)=e^{\alpha^{'}x}\times g\left( t;x,m,M \right)$ | (1) |
| --- | --- | --- |

## Separating the infection process from the clinical risk prediction

The model for the overall rate of death from COVID-19, Equation (1), is a proportional hazards model provided that $g(t;x,m,M)$ is not a function of x. This would be the case if neither the proportion of people-contacts infected nor the probability of being susceptible vary by patient characteristics. While both of these are likely to vary by geographical area, it is possible that within local geographical regions they may hold sufficiently well, suggesting that a Cox model stratified by region may be a reasonable model for COVID-19 death. However, it is often undesirable to include region in a risk prediction model because this restricts the potential generalisability of the model.

Therefore, whether it is possible to ignore the infection process and model only the relationship between patient characteristics and COVID-19 death depends on a number of assumptions about the dynamics of the disease process.

## Explicitly incorporating proxy measures of the burden of infection

The model for the rate of death from COVID-19 involves the function $f^{*}\left( j;x \right)$, related to the burden of infection, daily over the period (t-m, t-M). These measures are likely to be highly correlated; models are unlikely to fit well. Therefore, we fit a simpler model based on a quadratic approximation of the function $f^{*}\left( j;x \right)$ at each time-point.

days Suppose the function $f^{*}\left( j;x \right)$ (which reflects the combination of prevalence of infection on day j and the proportion of the population who are susceptible on that day) can be approximately modelled as a quadratic function of time, using data from days m to M prior to day t. For k=m,..,M,

$$f^{*}\left( t-k \right)\approx b_{t}-c_{t}k+d_{t}k^{2}$$

We can write $g\left( t;x,m,M \right)\approx\eta_{m}\left( b_{t}-c_{t}m+d_{t}m^{2} \right)+\ldots+ \eta_{M}(b_{t}-Mc_{t}+d_{t}M^{2})$

Which is $g\left( t;x,m,M \right)\approx{b_{t} \left( \eta_{m}+\ldots+ \eta_{M} \right)-c_{t}(m \eta}_{m}+\ldots+ M \eta_{M})+d_{t}(m^{2}\eta_{m}+\ldots{+M}^{2}\eta_{M})$

Or, parametrising the coefficients on the exponential scale, since they are constrained to be positive,

$$e^{B}=\left( \eta_{m}+\ldots+ \eta_{M} \right); e^{C}={(m \eta}_{m}+\ldots+ M \eta_{M})/e^{B}; e^{D}={(m^{2} \eta}_{m}+\ldots+M^{2}\eta_{M})/e^{B}$$

| Then | $g\left( t;x,m,M \right)\approx{e^{B}b}_{t}(1-e^{C}\frac{c_{t}}{b_{t}}{+e}^{D}\frac{d_{t}}{b_{t}} )$ |  |
| --- | --- | --- |

Substituting this into the model for the rate of COVID-19 death gives

|  | $\lambda\left( t \right)\approx\lambda_{0}\times e^{\alpha^{'}x}\times{e^{B}b}_{t}\times\left( 1-e^{C}\frac{c_{t}}{b_{t}}{+e}^{D}\frac{d_{t}}{b_{t}} \right)$ | (2) |
| --- | --- | --- |

On the log scale,

|  | $\log(\lambda(t))\approx cons+\alpha^{'}x+\log\left( b_{t} \right)+log\left( 1-e^{C}\frac{c_{t}}{b_{t}}{+e}^{D}\frac{d_{t}}{b_{t}} \right)$ | (3) |
| --- | --- | --- |

This suggests modelling the rate of COVID-19 death by a log-linear model including patient characteristics and the log of the quadratic coefficient $b_{t}$ (which is an estimate of $f^{*}\left( t;x \right)$). We approximate the last term by a second order polynomial in the two standardised quadratic coefficients (i.e. $\frac{c_{t}}{b_{t}}$ and $\frac{d_{t}}{b_{t}})$. Note that the term $e^{B}$ has been soaked up into the overall constant (cons).

Therefore, in the model selection for the functional form of the proxy measures of the burden of infection, we consider the log of the proxy measure and second-order polynomial terms of the standardised coefficients obtained from fitting a quadratic model to the proxy measure over the last three weeks (to provide an approximation to the last term in (3) above). We compare this to a range of alternative functional forms, including fractional polynomial models and cubic models of the proxy measure, using the AIC.

# Potential predictors

We selected candidate predictors based on known or plausible associations with exposure to COVID-19 infection, risk of severe illness or respiratory tract infection, and factors associated with healthcare access or level of care.

All codelists used to define predictors are listed in Table S1 below.

## Demographic and clinical predictors: additional details

Age (continuous); sex (male or female); e[thnicity](https://codelists.opensafely.org/codelist/opensafely/ethnicity/) (8 category: White, Indian, Pakistani, Bangladeshi/Other Asian, African/Other black, Caribbean, Chinese, Mixed/Other); deprivation (quintile of the index of multiple deprivation (IMD) derived from the patient’s postcode at lower super output area level); the number of adults living in the household (continuous) and whether or not children aged up to 12 years are living in the household (yes/no); and whether the individual lives in a rural or urban area were included.

Obesity was grouped using categories derived from the World Health Organisation classification of Body Mass Index (BMI; kg/m^2^): underweight <18.5 kg/m^2^; obese I 30-34.9; obese II 35-39.9; obese III 40+; or no evidence of obesity or being underweight, with BMI ascertained from weight measurements within the last 10 years, restricted to those taken when the patient was over 16 years old. Smoking status was grouped into evidence of current smoking in the last 18 months, former and never smokers.

Comorbidities were defined through combinations of clinical measurements, prescriptions, and recorded diagnoses. Blood pressure (in a measurement taken in the last 18 months) was grouped into: high II (systolic blood pressure (SBP) >= 140 mmHg or diastolic blood pressure (DBP) >= 90 mmHg), high I (SBP 130-<140 or DBP 80-<90), elevated (SPB 120-<130 and DBP <80) or normal (SBP<120 and DBP < 80). Where the two measures (SBP and DBP) differed, the higher (worse) class was taken. Diagnosed hypertension; [chronic cardiac disease](https://codelists.opensafely.org/codelist/opensafely/chronic-cardiac-disease/) including chronic heart failure, ischaemic heart disease, and severe valve or congenital heart disease likely to require lifelong follow up; atrial fibrillation; surgery for peripheral arterial disease or lower limb amputation; prior deep vein thrombosis or pulmonary embolism; [diabetes](https://codelists.opensafely.org/codelist/opensafely/diabetes/) (additionally using HbA1c within last 15 months to determine level of HbA1c control, grouped into <58 mmol/mol (good control), >=58 mmol/mol (poor control) and no recent measure); stroke; [dementia](https://codelists.opensafely.org/codelist/opensafely/dementia/); and o[ther neurological conditions](https://codelists.opensafely.org/codelist/opensafely/other-neurological-conditions/) (motor neurone disease, myasthenia gravis, multiple sclerosis, Parkinson's disease, cerebral palsy, quadriplegia or hemiplegia, malignant primary brain tumour, and progressive cerebellar disease) were included.

Asthma (grouped by use of oral corticosteroids as an indication of severity, with 2 or more prescriptions in the last year taken to indicate severe asthma); cystic fibrosis and associated diseases such as primary ciliary dyskinesia; and other r[espiratory disease](https://codelists.opensafely.org/codelist/opensafely/chronic-respiratory-disease/); haematological malignancies (considered separately from other cancers to reflect the immunosuppression associated with haematological malignancies and their treatment) and non-haematological malignancy, each grouped according to time since diagnosis (<1 year, 2-<5 years, 5+years); l[iver disease](https://codelists.opensafely.org/codelist/opensafely/chronic-liver-disease/); solid organ transplant; dialysis, for patients who have not since had a kidney transplant; and kidney function were included. Kidney function was ascertained from the most recent serum creatinine measurement taken in the last 5 years excluding the most recent fortnight, where available, converted into estimated glomerular filtration rate (eGFR) using the Chronic Kidney Disease Epidemiology Collaboration (CKD-EPI) equation), with reduced kidney function grouped into no evidence of kidney impairment (no creatinine measurement or eGFR>=60 mL/min/1.73m^2^), stage 3 (eGFR in range 30-<60 mL/min/1.73m^2^) and stage 4-5 (<30 mL/min/1.73m^2^). Patients with a history of kidney dialysis or kidney transplant were included in the category representing stage 4-5.

Rheumatoid Arthritis (RA), Systemic Lupus Erythematosus (SLE) or psoriasis; asplenia (splenectomy or a spleen dysfunction, including sickle cell disease); other immunosuppressive conditions including a condition inducing permanent immunodeficiency ever diagnosed, or aplastic anaemia or temporary immunodeficiency recorded within the last year; inflammatory bowel disease; HIV; learning disability, including Down’s syndrome; serious mental illness; and fragility fracture in the last two years for patients aged 65 or above were also included.

Information on the geographical region (seven regions of England: South West, South East, London, East, Midlands, North West, and North East, Yorkshire and the Humber) was available and used to undertake geographical internal validation.

## Proxy measures of the prevalence of COVID-19 infection: additional details

Three different proxy measures of infection prevalence, measured daily, were considered.

First, modelled estimates were obtained from dynamic disease modelling,^1^ with estimates obtained by region (7 regions in England) and by 5-year age-group. These estimates account for the infection prevalence, the way in which different age-groups interact with each other and the proportion of the population who are susceptible. These are estimates, thus come with uncertainty and potential error, neither of which is accounted for within our modelling.

Second, the mean daily rate of COVID-19 related A&E attendances over the last 7 days was estimated within each Sustainability and Transformation Partnership (STP; used as a measure of local geographic area). STP is a much finer measure of geographic area than region. Rate of A&E attendances is likely to be an imperfect proxy since it is likely to lag behind true prevalence of infection.

Third, the mean daily rate of suspected COVID-19 cases (with CTV3 Codes XaaNq, Y20cf, Y211b, Y22b7 and Y22b8 indicating a suspected case) in primary care over the last 7 days was estimated by STP. A&E attendances and suspected cases in primary care are both likely to lag behind the true infection prevalence, although this will not necessarily hinder performance in predicting COVID-19 mortality. They may also be sensitive to changes in how and when people interact with primary care providers.

## Predictors used in the simpler models

In the simpler model including only age and sex, the 10 age categories were: <40, 40-49, 50-59, 60-64, 65-69, 70-74, 75-79, 80-84, 85-89, 90+

The “number of comorbidities” model included demographic variables strongly linked to risk (age, sex, ethnicity, rurality), and a count of comorbidities previously found to be linked to COVID-19 mortality risk, see e.g. ^2^. Comorbidities included were: respiratory disease, cystic fibrosis, severe asthma (with OCS use), chronic cardiac disease, atrial fibrillation, deep vein thrombosis or pulmonary embolism, surgery for arterial disease, diabetes, cancer diagnosed in the last year, haematological malignancy diagnosed within 5 years, liver disease, stroke, dementia, neurological disease, poor kidney function (eGFR <60 mL/min/1.73m^2^), dialysis, organ transplant, asplenia, condition inducing immunosuppression, HIV, and either obese or underweight.

## Limitations in predictors for COVID-AGE

For the COVID-AGE risk tool, we could not reliably distinguish Type I from Type II diabetes so included all diabetes as Type II. We did not have an indicator of heart failure but included chronic cardiac disease.

## Details of selection of predictors

**For approach A**. Variable selection was performed within a 4% random sample of the whole eligible cohort. A Poisson model will be used for variable selection, using the whole 100-day period, with follow-up time accounted for via an offset term. Predictor variables were selected from the pool of candidates, by a lasso, with a penalty parameter chosen by 3-fold cross-validation. The set of possible parameters was specified using a logarithmic grid with 20 grid points. Continuous variables (age and number of people in the household) were standardised. Restricted cubic splines of the standardised variables were included. Sex and standardised age were forced into all models. Variables with non-zero coefficients were included in the subsequent models.

**For approach B**. Variable selection was undertaken separately for each proxy measure of infection prevalence. For each measure, we first selected the functional form of the time-varying measure of the proxy measure. This was done in a logistic model within the stacked case-cohort sub-studies, unadjusted for any other variables comparing different forms of the time-varying measures using the Akaike information criterion (AIC). For each time-varying proxy measure of the infection prevalence (model-based estimates, A&E attendance rate or suspected case rate), models considered for inclusion were: the current measure (evaluated as of day 0 of the relevant sub-study); the log of the current measure; the coefficients from a quadratic model of the measure fitted to the previous three weeks of data, expressed in relation to (i.e. divided by) the current measure; and polynomial terms of the current measure. Once the functional form of the time-varying measure of the proxy for infection prevalence was selected, variable selection was performed using a random sample from the whole cohort. Three non-overlapping 3% random samples of individuals from the whole eligible cohort were selected. Data from the 28-day period 1 March – 28 March (inclusive) were used for individuals in the first random sample; data from 6 April – 3 May (inclusive) for individuals in the second; and data from 12 May – 8 June for the third. Taking 28-day blocks from three non-overlapping periods, rather than all 73 landmark sub-studies for this step was intended to minimise effects of repeatedly including the same individuals on the variable selection process. Variable selection was performed within the three random samples, stacked to form one dataset, using a logistic regression lasso. The candidate pool was as above, with continuous variables and interactions treated as above. The chosen functional form of the relevant time-varying measure of the infection prevalence was be forced into the models. Variables with non-zero coefficients were included in the subsequent models.

Table S1. Codelists used to define candidate predictors and suspected COVID-19 (one of the proxy measures of infection burden)

| Variable | Notes | Codelist |
| --- | --- | --- |
| Suspected COVID-19 |  | <https://codelists.opensafely.org/codelist/opensafely/covid-identification-in-primary-care-suspected-covid-suspected-codes/2020-07-16/> |
| Ethnicity | 7 categories, obtained from 16 (White, Caribbean, Chinese, Indian/Pakistani, Mixed/Other, Bangladeshi/Other Asian, African/Other Black) | [https://codelists.opensafely.org/codelist/opensafely/ethnicity](https://codelists.opensafely.org/codelist/opensafely/ethnicity/) |
| Diagnosed hypertension |  | [https://codelists.opensafely.org/codelist/opensafely/hypertension](https://codelists.opensafely.org/codelist/opensafely/hypertension/) |
| C[hronic cardiac disease](https://codelists.opensafely.org/codelist/opensafely/chronic-cardiac-disease/) |  | <https://codelists.opensafely.org/codelist/opensafely/chronic-cardiac-disease/> |
| Atrial Fibrillation |  | <https://codelists.opensafely.org/codelist/opensafely/atrial-fibrillation-or-flutter/2020-07-30/> |
| Surgery for Peripheral Arterial Disease | Combined with lower limb amputation to form a peripheral arterial disease variable | <https://codelists.opensafely.org/codelist/opensafely/surgery-for-peripheral-artery-disease/2020-09-16/> |
| Lower limb amputation | Combined with surgery for peripheral arterial disease to form a peripheral arterial disease variable | <https://codelists.opensafely.org/codelist/opensafely/amputation/2020-09-21/> |
| Prior deep vein thrombosis / pulmonary embolism |  | <https://codelists.opensafely.org/codelist/opensafely/venous-thromboembolic-disease/2020-09-14/> |
| D[iabetes](https://codelists.opensafely.org/codelist/opensafely/diabetes/) | Combined with HbA1c measure within 18 months to determine level of control | <https://codelists.opensafely.org/codelist/opensafely/diabetes/> |
| S[troke](https://codelists.opensafely.org/codelist/opensafely/stroke-updated/) |  | <https://codelists.opensafely.org/codelist/opensafely/stroke/> |
| Dementia |  | <https://codelists.opensafely.org/codelist/opensafely/dementia/> |
| O[ther neurological conditions](https://codelists.opensafely.org/codelist/opensafely/other-neurological-conditions/) |  | <https://codelists.opensafely.org/codelist/opensafely/other-neurological-conditions/> |
| Asthma | Combined with OCS prescriptions in past year to determine severity | <https://codelists.opensafely.org/codelist/opensafely/asthma-diagnosis/> |
| Cystic Fibrosis and associated conditions |  | <https://codelists.opensafely.org/codelist/opensafely/cystic-fibrosis/2020-07-20/> |
| R[espiratory disease other than asthma](https://codelists.opensafely.org/codelist/opensafely/chronic-respiratory-disease/) or cystic fibrosis |  | <https://codelists.opensafely.org/codelist/opensafely/other-chronic-respiratory-disease/2020-07-20/> |
| Non-haematological cancer | Grouped by time since diagnosis (<1 year, 2-<5 years, 5+years) | <https://codelists.opensafely.org/codelist/opensafely/cancer-excluding-lung-and-haematological/> |
| Haematological cancer | Grouped by time since diagnosis (<1 year, 2-<5 years, 5+years) | <https://codelists.opensafely.org/codelist/opensafely/haematological-cancer/> |
| Lung cancer | Combined with other non-haematological cancer | <https://codelists.opensafely.org/codelist/opensafely/lung-cancer/> |
| Chronic l[iver disease](https://codelists.opensafely.org/codelist/opensafely/chronic-liver-disease/) |  | <https://codelists.opensafely.org/codelist/opensafely/chronic-liver-disease/> |
| Kidney dialysis | Used if no kidney transplant since most recent dialysis | <https://codelists.opensafely.org/codelist/opensafely/dialysis/2020-07-16/> |
| Kidney transplant | Combined with non-kidney transplant for transplant indicator. Also used to determine which of dialysis/transplant is most recent. | <https://codelists.opensafely.org/codelist/opensafely/kidney-transplant/2020-07-15/> |
| Organ transplant (other than kidney) | Combined with kidney transplant for transplant indicator. | <https://codelists.opensafely.org/codelist/opensafely/other-organ-transplant/2020-07-15/> |
| A[splenia](https://codelists.opensafely.org/codelist/opensafely/asplenia/) or dysplenia |  | <https://codelists.opensafely.org/codelist/opensafely/asplenia/> <https://codelists.opensafely.org/codelist/opensafely/sickle-cell-disease/> |
| Autoimmune diseases ([rheumatoid arthritis, lupus, psoriasis](https://codelists.opensafely.org/codelist/opensafely/ra-sle-psoriasis/)) |  | <https://codelists.opensafely.org/codelist/opensafely/ra-sle-psoriasis/> |
| HIV |  | [https://codelists.opensafely.org/codelist/opensafely/hiv/2020-07-13/](https://codelists.opensafely.org/codelist/opensafely/hiv/2020-07-13/#full-list) |
| Other immunosuppressive condition | Temporary and aplastic anaemia within last year; permanent ever. | <https://codelists.opensafely.org/codelist/opensafely/permanent-immunosuppresion/>  <https://codelists.opensafely.org/codelist/opensafely/aplastic-anaemia/>  <https://codelists.opensafely.org/codelist/opensafely/temporary-immunosuppresion/> |
| Inflammatory bowel disease |  | <https://codelists.opensafely.org/codelist/opensafely/inflammatory-bowel-disease/2020-04-07/> |
| Learning disability, including Down’s syndrome |  | <https://codelists.opensafely.org/codelist/opensafely/intellectual-disability-including-downs-syndrome/2020-08-27/> |
| Serious mental illness |  | <https://codelists.opensafely.org/codelist/opensafely/psychosis-schizophrenia-bipolar-affective-disease/2020-07-09/> |
| Fragility fracture |  | <https://codelists.opensafely.org/codelist/opensafely/fragility/2020-09-14/> |

# Additional sensitivity analyses undertake

## Comparing the cohort and case-cohort design

For approach A, the Cox model was refitted in the whole cohort, to assess robustness of results to the design chosen. The model coefficients obtained from the model fitted on the whole cohort were used to predict the outcome in each validation cohort; measures of model performance were assessed as for the main validation.

## Additional model types

For approach A, Royston-Parmar, Weibull and Generalised gamma models were also fitted.

For approach B, Weibull and logistic models were also fitted in addition to the Poisson model of the main text.

Results from approach A and B both using Weibull models were compared, to remove the difference in model format from the comparison.

## Combining proxy measures of the burden of COVID-19 infection

Rather than fit separate models for each of the three proxy measures of the burden of infection, we could simultaneously include these measures in the same model. We additionally fitted models including all three measures, and models including the two objective measures able to be obtained through routinely collected data – A&E rates and suspected case rates in primary care. In each case, the predictors included were the union of the covariate set selected for models including only the individual included proxies of burden of infection.

## Not updating patient characteristics in landmark sub-studies

In the main approach B (landmarking) modes, patient demographic and comorbidity data were updated through time. For each sub-study the predictors were measured as of day 0. In a sensitivity analysis, we kept these predictors fixed from cohort entry.

## Removing the proxies of burden of infection

For approach B, we additionally fitted models which did not include any information related to the proxies of burden of COVID-19 infection, to assess the effect of including those.

# Study flowchart

Figure S1. Flowchart of patients through study selection.


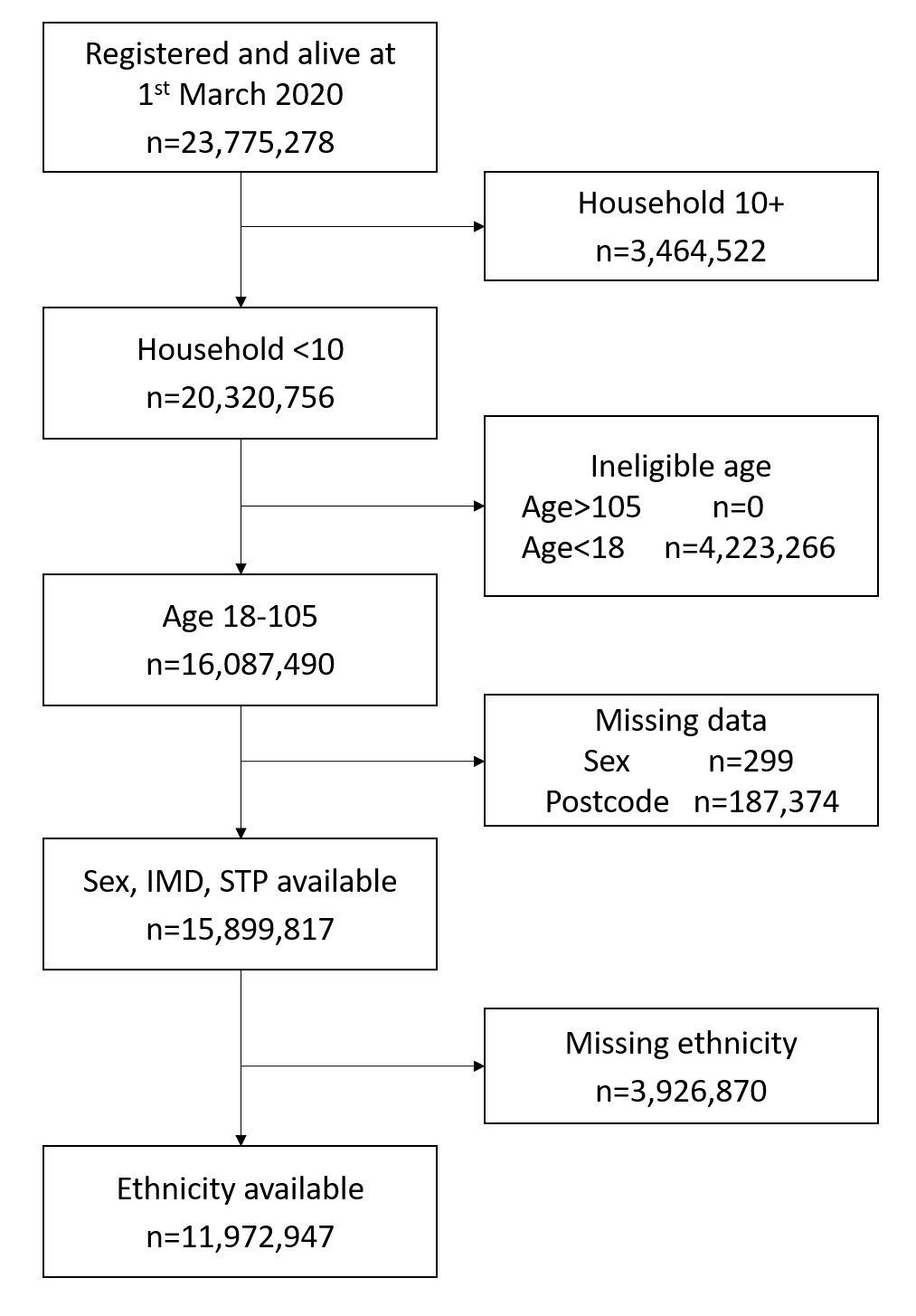


# Estimated regression coefficients

Table S2. Estimated Hazard Ratios from the “selected” Cox model, approach A

| Characteristic | Sub-distribution Hazard Ratio (95% CI) |
| --- | --- |
| Age (per unit*) | 7.56 (7.16, 7.98) |
| Male | 2.13 (1.97, 2.29) |
| Urban | 1.52 (1.42, 1.62) |
| IMD 1 (least deprived) | 0.79 (0.74, 0.84) |
| IMD 5 (most deprived) | 1.33 (1.22, 1.45) |
| Ethnicity: White | 0.58 (0.54, 0.63) |
| BMI: Underweight | 1.53 (1.33, 1.75) |
| BMI: Normal/overweight | 0.81 (0.77, 0.86) |
| Diabetes: None | 0.68 (0.64, 0.72) |
| Diabetes: HbA1c unknown | 1.22 (1.07, 1.39) |
| No stroke | 0.71 (0.64, 0.79) |
| No dementia | 0.36 (0.30, 0.42) |
| No other neurological condition | 0.14 (0.10, 0.18) |
| Asthma: With OCS use | 1.23 (1.06, 1.43) |
| No respiratory disease | 0.35 (0.28, 0.42) |
| Cancer (exc. haematological): Last year | 1.63 (1.34, 2.00) |
| Cancer (haematological): Never | 0.44 (0.37, 0.53) |
| No liver disease | 0.27 (0.17, 0.41) |
| Renal impairment: None | 0.70 (0.65, 0.76) |
| Renal impairment: Stage 4/5 | 10.08 (7.24, 14.03) |
| No immunosuppression | 0.47 (0.30, 0.74) |
| No serious mental illness | 0.41 (0.36, 0.48) |
| *Interactions with age***:* |  |
| BMI: Obese II (Per unit age increase) | 1.14 (1.06, 1.21) |
| BMI: Obese III (Per unit age increase) | 0.94 (0.79, 1.12) |
| Current smoker (Per unit age increase) | 0.97 (0.91, 1.03) |
| Diagnosed hypertension (Per unit age increase) | 0.98 (0.95, 1.01) |
| Diabetes: Uncontrolled (Per unit age increase) | 0.99 (0.92, 1.07) |
| Other neurological condition (Per unit age increase) | 0.51 (0.43, 0.61) |
| Respiratory disease (Per unit age increase) | 0.70 (0.62, 0.78) |
| Cancer (haematological): Last year (Per unit age increase) | 1.07 (0.78, 1.46) |
| Liver disease (Per unit age increase) | 0.62 (0.46, 0.85) |
| Dialysis (Per unit age increase) | 1.45 (1.17, 1.80) |
| Renal impairment: Stage 4/5 (Per unit age increase) | 0.37 (0.31, 0.44) |
| Asplenia (Per unit age increase) | 1.01 (0.77, 1.33) |
| Inflammatory bowel disease (Per unit age increase) | 1.11 (1.00, 1.23) |
| Fracture (Per unit age increase) | 1.25 (1.16, 1.34) |
| *Interactions with sex:* |  |
| (In males) IMD 5 (most deprived) | 0.97 (0.86, 1.08) |
| (In females) BMI: Obese III | 1.83 (1.36, 2.48) |
| (In males) Diabetes: Uncontrolled | 1.39 (1.19, 1.62) |
| (In females) Cardiac disease | 1.30 (1.19, 1.41) |
| (In females) Atrial Fibrillation | 1.22 (1.11, 1.34) |
| (In males) Surgery for peripheral arterial disease | 1.72 (1.41, 2.09) |
| (In males) Stroke | 1.05 (0.91, 1.21) |
| (In males) Dementia | 1.22 (0.97, 1.54) |
| (In females) Asthma: Without OCS use | 1.07 (0.96, 1.18) |
| (In females) Respiratory disease | 1.26 (1.12, 1.42) |
| (In females) Cancer (exc. haematological): Last year | 2.01 (1.49, 2.72) |
| (In males) Cancer (exc. haematological): 2-5 years ago | 1.25 (1.09, 1.44) |
| (In males) Cancer (exc. haematological): 5+ years ago | 1.06 (0.96, 1.17) |
| (In females) Cancer (haematological): Last year | 1.28 (0.54, 3.02) |
| (In males) Cancer (haematological): 5+ years ago | 0.60 (0.44, 0.82) |
| (In females) Liver disease | 1.27 (0.88, 1.82) |
| (In females) Dialysis | 1.61 (0.98, 2.63) |
| (In females) Renal impairment: Stage 4/5 | 1.25 (1.02, 1.54) |
| (In males) Renal impairment: Stage 3a/3b | 0.96 (0.86, 1.07) |
| (In males) RA/SLE/Psoriasis | 1.12 (1.00, 1.26) |
| (In males) Fracture | 1.65 (1.32, 2.06) |
|  | Estimated baseline survival |
| At 28 days | 0.9873567 |

* Age measured in SD units (1 unit = 18.84 years).

Abbreviations: BMI Body Mass Index; IMD Index of Multiple Deprivation; OCS Oral corticosteroids; RA rheumatoid arthritis; SLE Systemic lupus erythematosus.

Table S3: Estimated Incidence Rate Ratios from the “selected” Poisson model, approach B, incorporating the modelled estimates as a proxy for the infection prevalence

| Characteristic | Incidence Rate Ratio (95% CI) |
| --- | --- |
| *Related to modelled estimates (infection prevalence proxy)* |  |
| Log of modelled estimate | 1.91 (1.85,1.97) |
| Standardised quadratic coefficient* | 7.98 (3.79,16.83) |
| *Patient characteristics* |  |
| Age (per unit**) | 7.66 (7.24,8.10) |
| Male | 2.13 (1.97, 2.31) |
| Urban | 1.41 (1.27, 1.56) |
| IMD 1 (least deprived) | 0.47 (0.39, 0.56) |
| Ethnicity: Mixed | 1.23 (1.02, 1.47) |
| BMI: Obese II | 2.05 (1.69, 2.49) |
| Diabetes: None | 0.67 (0.63, 0.71) |
| Diabetes: Uncontrolled | 1.52 (1.37, 1.69) |
| No cardiac disease | 0.79 (0.75, 0.84) |
| No deep vein thrombosis or pulmonary embolism | 0.64 (0.59, 0.70) |
| No other neurological condition | 0.44 (0.38, 0.51) |
| No respiratory disease | 0.59 (0.54, 0.64) |
| Cancer (exc. haematological): Last year | 10.47 (7.22, 15.18) |
| No organ transplant | 0.47 (0.31, 0.72) |
| Renal impairment: None | 0.67 (0.61, 0.73) |
| Renal impairment: Stage 4/5 | 1.89 (1.61, 2.21) |
| No intellectual disability | 0.23 (0.19, 0.28) |
| No fracture | 0.49 (0.42, 0.56) |
| Number in household (spline term 2***) | 2.53 (1.59,4.02) |
| Number in household (spline term 3***) | 0.19 (0.05,0.68) |
| *Interactions with age**:* |  |
| IMD 1 (least deprived; Per unit age increase) | 1.38 (1.24, 1.53) |
| IMD 5 (most deprived; Per unit age increase) | 1.13 (1.08, 1.19) |
| Ethnicity: Indian (Per unit age increase) | 0.98 (0.90, 1.08) |
| Ethnicity: Pakistani (Per unit age increase) | 0.88 (0.77, 1.00) |
| BMI: Obese II (Per unit age increase) | 0.79 (0.69, 0.91) |
| BMI: Obese III (Per unit age increase) | 1.06 (0.88, 1.26) |
| Blood pressure: Normal (Per unit age increase) | 1.23 (1.18, 1.28) |
| Blood pressure: Stage I (Per unit age increase) | 0.95 (0.92, 0.98) |
| Atrial Fibrillation (Per unit age increase) | 1.09 (1.04, 1.14) |
| Surgery for peripheral arterial disease (Per unit age increase) | 1.30 (1.18, 1.42) |
| Stroke (Per unit age increase) | 1.08 (1.03, 1.15) |
| Dementia (Per unit age increase) | 1.48 (1.35, 1.62) |
| Asthma: Without OCS use (Per unit age increase) | 0.97 (0.92, 1.01) |
| Asthma: With OCS use (Per unit age increase) | 0.98 (0.85, 1.12) |
| Cancer (exc. haematological): Last year (Per unit age increase) | 0.35 (0.28, 0.43) |
| Cancer (exc. haematological): 2-5 years ago (Per unit age increase) | 1.12 (1.05, 1.21) |
| Cancer (exc. haematological): 5+ years ago (Per unit age increase) | 1.01 (0.96, 1.05) |
| Cancer (haematological): 5+ years ago (Per unit age increase) | 1.20 (1.06, 1.35) |
| Dialysis (Per unit age increase) | 0.43 (0.29, 0.63) |
| Asplenia (Per unit age increase) | 1.02 (0.77, 1.35) |
| Serious mental illness (Per unit age increase) | 1.42 (1.19, 1.69) |
| *Interactions with sex:* |  |
| (In males) Rural | 0.87 (0.76, 1.00) |
| (In females) IMD 5 (most deprived) | 1.06 (0.94, 1.19) |
| (In females) BMI: Obese III | 1.68 (1.24, 2.29) |
| (In females) Diabetes: Uncontrolled | 1.01 (0.86, 1.19) |
| (In females) Atrial Fibrillation | 1.03 (0.90, 1.17) |
| (In females) Stroke | 1.18 (1.01, 1.39) |
| (In males) Dementia | 1.52 (1.18, 1.96) |
| (In females) Other neurological condition | 1.24 (0.97, 1.57) |
| (In females) Asthma: With OCS use | 2.26 (1.70, 3.01) |
| (In females) Respiratory | 1.19 (1.05, 1.35) |
| (In females) Cancer (exc. haematological): Last year | 1.76 (1.29, 2.40) |
| (In males) Cancer (haematological): Last year | 3.26 (1.97, 5.41) |
| (In males) Cancer (haematological): 2-5 years ago | 2.61 (1.96, 3.48) |
| (In females) Dialysis | 14.60 (7.65, 27.89) |
| (In males) Dialysis | 7.24 (3.83, 13.68) |
| (In males) Renal impairment: Stage 3a/3b | 0.88 (0.79, 0.99) |
| (In males) Renal impairment: Stage 4/5 | 0.82 (0.66, 1.01) |
| (In females) Inflammatory bowel disease | 1.18 (0.91, 1.54) |
| (In females) Serious mental illness | 1.31 (0.91, 1.89) |
| (In males) Fracture | 1.36 (1.09, 1.70) |
| Constant | 0.01075 (0.00595, 0.01942) |

* Standardised quadratic coefficient obtained by fitting a quadratic model to the last three weeks of the measure, and dividing the linear coefficient by the constant coefficient.

** Age measured in SD units (1 unit = 18.84 years).

*** Household splines can be obtained using the following centiles: p_5_=1 p_35_=2 p_65_=3 p_95_=6.

Abbreviations: BMI Body Mass Index; IMD Index of Multiple Deprivation; OCS Oral corticosteroids; RA rheumatoid arthritis; SLE Systemic lupus erythematosus.

# Model performance: internal validation

Table S4 shows model performance of the “selected” models separately by geographical region.

Table S5 shows C-statistics for different sets of predictor variables, by sex and broad age-group for approach B using proxy measures using A&E and GP data. Analogous statistics for approach A and approach B using modelled estimates can be found in the main text.

Figure S2 graphs the C-statistics for different sets of predictor variables, by sex and broad age-group, for all approaches.

Figures S3-S6 show flexible calibration curves and predicted vs observed risk by twentieths of predicted risk for the “selected” models.

Table S4. Measures of model performance (“selected” model) in predicting 28-day risk of COVID-19 mortality, by region

| Measures of infection prevalence included (approach) and model form | Validation  period | Region | C-statistic | Observed mean risk (%) | Predicted mean risk (%) | Calibration | |
| --- | --- | --- | --- | --- | --- | --- | --- |
|  |  |  |  |  |  | Intercept (95% CI) | Slope (95% CI) |
| None (A),  Cox | 1 | East | 0.909 | 0.0036 | 0.0034 | 0.04 (-0.15, 0.24) | 0.87 (0.67, 1.07) |
|  |  | London | 0.959 | 0.0102 | 0.0034 | 1.12 (0.92, 1.31) | 1.04 (0.84, 1.23) |
|  |  | Midlands | 0.928 | 0.0052 | 0.0041 | 0.23 (0.06, 0.39) | 0.97 (0.80, 1.13) |
|  |  | North East/Yorks | 0.903 | 0.0024 | 0.0041 | -0.54 (-0.81, -0.27) | 0.85 (0.59, 1.12) |
|  |  | North West | 0.838 | 0.0021 | 0.0043 | -0.73 (-1.15, -0.31) | 0.65 (0.24, 1.07) |
|  |  | South East | 0.952 | 0.0026 | 0.0034 | -0.28 (-0.73, 0.17) | 1.10 (0.66, 1.55) |
|  |  | South West | 0.936 | 0.0014 | 0.0038 | -1.00 (-1.43, -0.57) | 1.00 (0.57, 1.43) |
|  | 2 | East | 0.932 | 0.0365 | 0.0034 | 2.39 (2.32, 2.45) | 1.05 (0.99, 1.11) |
|  |  | London | 0.941 | 0.0396 | 0.0033 | 2.49 (2.39, 2.59) | 0.92 (0.82, 1.01) |
|  |  | Midlands | 0.932 | 0.0405 | 0.0041 | 2.30 (2.24, 2.36) | 1.01 (0.95, 1.07) |
|  |  | North East/Yorks | 0.935 | 0.0420 | 0.0041 | 2.33 (2.27, 2.4) | 1.01 (0.95, 1.07) |
|  |  | North West | 0.928 | 0.0493 | 0.0043 | 2.45 (2.36, 2.53) | 1.03 (0.95, 1.12) |
|  |  | South East | 0.937 | 0.0238 | 0.0034 | 1.96 (1.81, 2.11) | 1.04 (0.90, 1.19) |
|  |  | South West | 0.938 | 0.0235 | 0.0037 | 1.84 (1.73, 1.94) | 1.07 (0.97, 1.17) |
|  | 3 | East | 0.941 | 0.0106 | 0.0033 | 1.16 (1.05, 1.28) | 1.08 (0.97, 1.20) |
|  |  | London | 0.955 | 0.0033 | 0.0033 | 0.02 (-0.31, 0.36) | 0.96 (0.63, 1.30) |
|  |  | Midlands | 0.935 | 0.0120 | 0.0040 | 1.10 (0.99, 1.21) | 1.00 (0.89, 1.11) |
|  |  | North East/Yorks | 0.946 | 0.0142 | 0.0040 | 1.26 (1.15, 1.37) | 1.07 (0.96, 1.18) |
|  |  | North West | 0.937 | 0.0168 | 0.0042 | 1.38 (1.23, 1.52) | 1.08 (0.93, 1.23) |
|  |  | South East | 0.962 | 0.0053 | 0.0033 | 0.46 (0.14, 0.77) | 1.22 (0.91, 1.54) |
|  |  | South West | 0.918 | 0.0047 | 0.0037 | 0.24 (0.01, 0.48) | 1.06 (0.82, 1.29) |

Table S4 ctd. Measures of model performance (“selected” model) in predicting 28-day risk of COVID-19 mortality, by region

| Measures of infection prevalence included (approach) and model form | Validation  period | Region | C-statistic | Observed mean risk (%) | Predicted mean risk (%) | Calibration | |
| --- | --- | --- | --- | --- | --- | --- | --- |
|  |  |  |  |  |  | Intercept (95% CI) | Slope (95% CI) |
| Modelled  estimates (B),  Poisson | 1 | East | 0.914 | 0.0036 | 0.0025 | 0.34 (0.14, 0.54) | 0.88 (0.68, 1.07) |
|  |  | London | 0.954 | 0.0102 | 0.0081 | 0.23 (0.04, 0.42) | 1.01 (0.82, 1.20) |
|  |  | Midlands | 0.924 | 0.0052 | 0.0058 | -0.12 (-0.29, 0.05) | 0.92 (0.75, 1.09) |
|  |  | North East/Yorks | 0.907 | 0.0024 | 0.0035 | -0.39 (-0.65, -0.12) | 0.86 (0.59, 1.13) |
|  |  | North West | 0.860 | 0.0021 | 0.0045 | -0.77 (-1.19, -0.36) | 0.72 (0.31, 1.14) |
|  |  | South East | 0.938 | 0.0026 | 0.0047 | -0.60 (-1.05, -0.15) | 1.00 (0.55, 1.45) |
|  |  | South West | 0.931 | 0.0014 | 0.0039 | -1.03 (-1.45, -0.6) | 0.93 (0.50, 1.36) |
|  | 2 | East | 0.936 | 0.0365 | 0.0355 | 0.03 (-0.03, 0.09) | 1.03 (0.96, 1.09) |
|  |  | London | 0.937 | 0.0396 | 0.0396 | 0.00 (-0.10, 0.10) | 0.93 (0.83, 1.02) |
|  |  | Midlands | 0.934 | 0.0405 | 0.0376 | 0.08 (0.02, 0.14) | 1.00 (0.94, 1.06) |
|  |  | North East/Yorks | 0.939 | 0.0420 | 0.0362 | 0.15 (0.09, 0.21) | 1.00 (0.94, 1.07) |
|  |  | North West | 0.931 | 0.0493 | 0.0402 | 0.21 (0.12, 0.29) | 1.00 (0.91, 1.08) |
|  |  | South East | 0.943 | 0.0238 | 0.0314 | -0.28 (-0.43, -0.13) | 1.04 (0.89, 1.19) |
|  |  | South West | 0.941 | 0.0235 | 0.0257 | -0.09 (-0.19, 0.02) | 1.02 (0.92, 1.13) |
|  | 3 | East | 0.944 | 0.0106 | 0.0101 | 0.05 (-0.07, 0.16) | 1.05 (0.94, 1.17) |
|  |  | London | 0.954 | 0.0033 | 0.0100 | -1.09 (-1.43, -0.76) | 0.94 (0.61, 1.28) |
|  |  | Midlands | 0.938 | 0.0120 | 0.0156 | -0.26 (-0.37, -0.15) | 0.99 (0.88, 1.10) |
|  |  | North East/Yorks | 0.944 | 0.0142 | 0.0150 | -0.05 (-0.16, 0.06) | 1.04 (0.93, 1.15) |
|  |  | North West | 0.936 | 0.0168 | 0.0209 | -0.22 (-0.37, -0.07) | 1.04 (0.89, 1.19) |
|  |  | South East | 0.965 | 0.0053 | 0.0108 | -0.72 (-1.03, -0.40) | 1.16 (0.85, 1.47) |
|  |  | South West | 0.922 | 0.0047 | 0.0065 | -0.33 (-0.56, -0.09) | 1.04 (0.81, 1.27) |

Table S4 ctd. Measures of model performance (“selected” model) in predicting 28-day risk of COVID-19 mortality, by region

| Measures of infection prevalence included (approach) and model form | Validation  period | Region | C-statistic | Observed mean risk (%) | Predicted mean risk (%) | Calibration | |
| --- | --- | --- | --- | --- | --- | --- | --- |
|  |  |  |  |  |  | Intercept (95% CI) | Slope (95% CI) |
| A&E COVID-19  attendances  (B),  Poisson | 1 | East | 0.913 | 0.0036 | 0.0132 | -1.32 (-1.51, -1.12) | 0.88 (0.68, 1.08) |
|  |  | London | 0.950 | 0.0102 | 0.0118 | -0.14 (-0.33, 0.05) | 1.01 (0.81, 1.20) |
|  |  | Midlands | 0.924 | 0.0052 | 0.0153 | -1.09 (-1.26, -0.92) | 0.93 (0.77, 1.10) |
|  |  | North East/Yorks | 0.909 | 0.0024 | 0.0155 | -1.87 (-2.13, -1.60) | 0.85 (0.58, 1.11) |
|  |  | North West | 0.846 | 0.0021 | 0.0166 | -2.08 (-2.49, -1.66) | 0.69 (0.27, 1.11) |
|  |  | South East | 0.947 | 0.0026 | 0.0132 | -1.65 (-2.10, -1.20) | 1.05 (0.60, 1.50) |
|  |  | South West | 0.936 | 0.0014 | 0.0149 | -2.37 (-2.80, -1.95) | 0.97 (0.54, 1.39) |
|  | 2 | East | 0.934 | 0.0365 | 0.0426 | -0.16 (-0.22, -0.09) | 1.04 (0.97, 1.10) |
|  |  | London | 0.937 | 0.0396 | 0.0243 | 0.50 (0.40, 0.59) | 0.93 (0.83, 1.03) |
|  |  | Midlands | 0.932 | 0.0405 | 0.0552 | -0.31 (-0.37, -0.25) | 0.98 (0.92, 1.04) |
|  |  | North East/Yorks | 0.935 | 0.0420 | 0.0378 | 0.11 (0.04, 0.17) | 1.00 (0.93, 1.06) |
|  |  | North West | 0.927 | 0.0493 | 0.0542 | -0.1 (-0.18, -0.01) | 0.99 (0.90, 1.08) |
|  |  | South East | 0.939 | 0.0238 | 0.0259 | -0.08 (-0.23, 0.06) | 1.06 (0.91, 1.20) |
|  |  | South West | 0.940 | 0.0235 | 0.0348 | -0.4 (-0.50, -0.29) | 1.05 (0.94, 1.15) |
|  | 3 | East | 0.943 | 0.0106 | 0.0182 | -0.54 (-0.66, -0.43) | 1.07 (0.95, 1.18) |
|  |  | London | 0.948 | 0.0033 | 0.0147 | -1.48 (-1.82, -1.15) | 0.94 (0.61, 1.28) |
|  |  | Midlands | 0.938 | 0.0120 | 0.0230 | -0.65 (-0.76, -0.54) | 1.01 (0.9, 1.12) |
|  |  | North East/Yorks | 0.944 | 0.0142 | 0.0227 | -0.47 (-0.58, -0.36) | 1.05 (0.94, 1.16) |
|  |  | North West | 0.937 | 0.0168 | 0.0213 | -0.24 (-0.39, -0.09) | 1.03 (0.89, 1.18) |
|  |  | South East | 0.966 | 0.0053 | 0.0122 | -0.85 (-1.16, -0.53) | 1.18 (0.87, 1.50) |
|  |  | South West | 0.928 | 0.0047 | 0.0179 | -1.34 (-1.57, -1.11) | 1.09 (0.86, 1.32) |

Table S4 ctd. Measures of model performance (“selected” model) in predicting 28-day risk of COVID-19 mortality, by region

| Measures of infection prevalence included (approach) and model form | Validation  period | Region | C-statistic | Observed mean risk (%) | Predicted mean risk (%) | Calibration | |
| --- | --- | --- | --- | --- | --- | --- | --- |
|  |  |  |  |  |  | Intercept (95% CI) | Slope (95% CI) |
| Suspected  COVID-19 in  primary care  (B),  Poisson | 1 | East | 0.913 | 0.0036 | 0.0092 | -0.95 (-1.15, -0.75) | 0.88 (0.68, 1.07) |
|  |  | London | 0.954 | 0.0102 | 0.0073 | 0.34 (0.15, 0.53) | 1.02 (0.82, 1.21) |
|  |  | Midlands | 0.918 | 0.0052 | 0.0085 | -0.5 (-0.66, -0.33) | 0.89 (0.72, 1.05) |
|  |  | North East/Yorks | 0.911 | 0.0024 | 0.0090 | -1.33 (-1.59, -1.06) | 0.84 (0.57, 1.11) |
|  |  | North West | 0.860 | 0.0021 | 0.0047 | -0.82 (-1.24, -0.40) | 0.72 (0.3, 1.13) |
|  |  | South East | 0.936 | 0.0026 | 0.0094 | -1.3 (-1.75, -0.85) | 1.00 (0.55, 1.45) |
|  |  | South West | 0.932 | 0.0014 | 0.0092 | -1.89 (-2.32, -1.47) | 0.93 (0.50, 1.36) |
|  | 2 | East | 0.936 | 0.0365 | 0.0341 | 0.07 (0.01, 0.13) | 1.03 (0.96, 1.09) |
|  |  | London | 0.938 | 0.0396 | 0.0299 | 0.28 (0.19, 0.38) | 0.93 (0.83, 1.02) |
|  |  | Midlands | 0.933 | 0.0405 | 0.0398 | 0.02 (-0.04, 0.08) | 1.00 (0.94, 1.06) |
|  |  | North East/Yorks | 0.938 | 0.0420 | 0.0414 | 0.01 (-0.05, 0.08) | 1.01 (0.94, 1.07) |
|  |  | North West | 0.927 | 0.0493 | 0.0410 | 0.19 (0.10, 0.27) | 0.98 (0.89, 1.07) |
|  |  | South East | 0.942 | 0.0238 | 0.0354 | -0.4 (-0.55, -0.25) | 1.04 (0.89, 1.19) |
|  |  | South West | 0.940 | 0.0235 | 0.0396 | -0.53 (-0.63, -0.42) | 1.02 (0.92, 1.13) |
|  | 3 | East | 0.943 | 0.0106 | 0.0152 | -0.36 (-0.47, -0.24) | 1.06 (0.94, 1.17) |
|  |  | London | 0.951 | 0.0033 | 0.0079 | -0.86 (-1.20, -0.53) | 0.93 (0.59, 1.26) |
|  |  | Midlands | 0.937 | 0.0120 | 0.0175 | -0.38 (-0.49, -0.27) | 0.99 (0.88, 1.10) |
|  |  | North East/Yorks | 0.943 | 0.0142 | 0.0184 | -0.26 (-0.37, -0.15) | 1.05 (0.94, 1.16) |
|  |  | North West | 0.936 | 0.0168 | 0.0149 | 0.12 (-0.03, 0.27) | 1.04 (0.90, 1.19) |
|  |  | South East | 0.963 | 0.0053 | 0.0156 | -1.09 (-1.41, -0.78) | 1.13 (0.82, 1.45) |
|  |  | South West | 0.921 | 0.0047 | 0.0147 | -1.14 (-1.37, -0.91) | 1.06 (0.82, 1.29) |

Table S5. C-statistics for different sets of predictor variables, by sex and broad age-group for approach B using A&E attendance and primary care data as proxy measures of infection prevalence

| Measures of infection  prevalence included (approach)  and model form | Validation  period | Predictor set | **C-statistic** | | | | | |
| --- | --- | --- | --- | --- | --- | --- | --- | --- |
|  |  |  | **Age 18-<70** | | **Age 70-<80** | | **Age 80+** | |
|  |  |  | **Female** | **Male** | **Female** | **Male** | **Female** | **Male** |
| A&E COVID-19  attendances (B),  Poisson | 1 | Age-sex | 0.81 | 0.83 | 0.58 | 0.58 | 0.64 | 0.53 |
|  |  | Comorbidities | 0.89 | 0.92 | 0.84 | 0.77 | 0.77 | 0.63 |
|  |  | Selected | 0.86 | 0.90 | 0.83 | 0.78 | 0.75 | 0.65 |
|  |  | Full | 0.89 | 0.93 | 0.85 | 0.79 | 0.76 | 0.66 |
|  | 2 | Age-sex | 0.80 | 0.82 | 0.63 | 0.58 | 0.64 | 0.65 |
|  |  | Comorbidities | 0.90 | 0.89 | 0.78 | 0.73 | 0.71 | 0.70 |
|  |  | Selected | 0.88 | 0.88 | 0.83 | 0.77 | 0.77 | 0.75 |
|  |  | Full | 0.92 | 0.91 | 0.84 | 0.79 | 0.78 | 0.76 |
|  | 3 | Age-sex | 0.85 | 0.83 | 0.62 | 0.63 | 0.69 | 0.68 |
|  |  | Comorbidities | 0.91 | 0.89 | 0.79 | 0.75 | 0.72 | 0.72 |
|  |  | Selected | 0.91 | 0.89 | 0.85 | 0.80 | 0.78 | 0.76 |
|  |  | Full | 0.92 | 0.91 | 0.86 | 0.83 | 0.79 | 0.79 |
| Suspected COVID-19  in primary care (B),  Poisson | 1 | Age-sex | 0.80 | 0.84 | 0.57 | 0.60 | 0.64 | 0.55 |
|  |  | Comorbidities | 0.88 | 0.92 | 0.83 | 0.78 | 0.76 | 0.64 |
|  |  | Selected | 0.85 | 0.91 | 0.82 | 0.76 | 0.75 | 0.66 |
|  |  | Full | 0.88 | 0.93 | 0.84 | 0.79 | 0.75 | 0.66 |
|  | 2 | Age-sex | 0.79 | 0.81 | 0.58 | 0.55 | 0.63 | 0.62 |
|  |  | Comorbidities | 0.89 | 0.89 | 0.77 | 0.73 | 0.70 | 0.68 |
|  |  | Selected | 0.89 | 0.89 | 0.82 | 0.78 | 0.76 | 0.74 |
|  |  | Full | 0.92 | 0.91 | 0.83 | 0.80 | 0.77 | 0.76 |
|  | 3 | Age-sex | 0.84 | 0.82 | 0.59 | 0.62 | 0.66 | 0.66 |
|  |  | Comorbidities | 0.91 | 0.89 | 0.79 | 0.74 | 0.71 | 0.71 |
|  |  | Selected | 0.90 | 0.88 | 0.84 | 0.81 | 0.77 | 0.76 |
|  |  | Full | 0.92 | 0.90 | 0.86 | 0.83 | 0.78 | 0.78 |

Figure S2. C-statistics for different sets of predictor variables, by sex and broad age-group for all approached. The three dots of the same colours represent the values for the three validation periods (exact values provided in Tables 3 (main text) and S5). Axis labels indicate the approach: ME modelled estimates, AE A&E COVID-19 attendances, GP suspected COVID-19 in primary care.

Figure S3: Approach A “selected” model – flexible calibration curves (top) and observed vs predicted COVID-19 mortality by twentieths of predicted risk for each validation period (VP 1, 2 and 3).


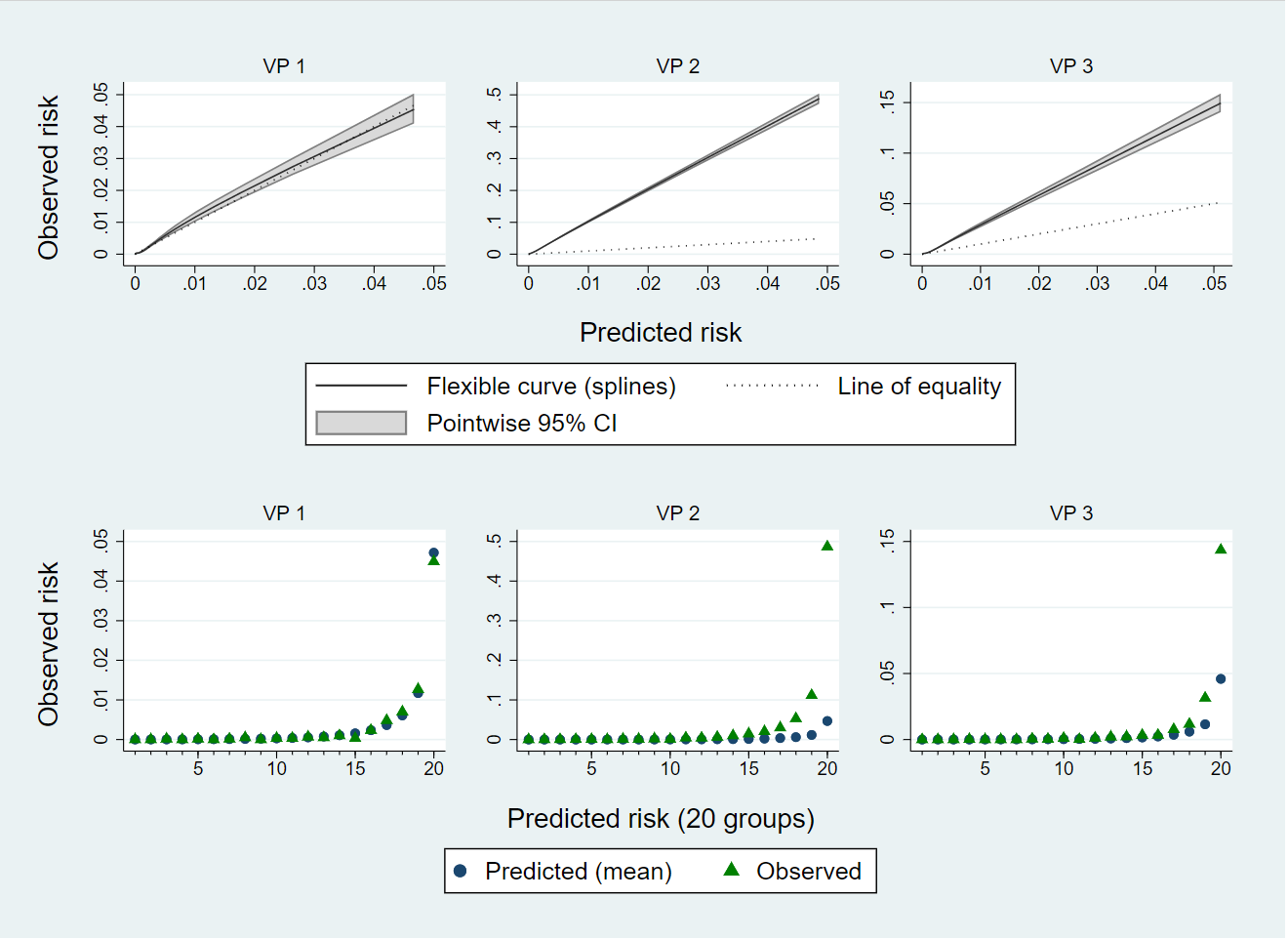


Figure S4: Approach B “selected” model including modelled estimates – flexible calibration curves (top) and observed vs predicted COVID-19 mortality by twentieths of predicted risk for each validation period (VP 1, 2 and 3).


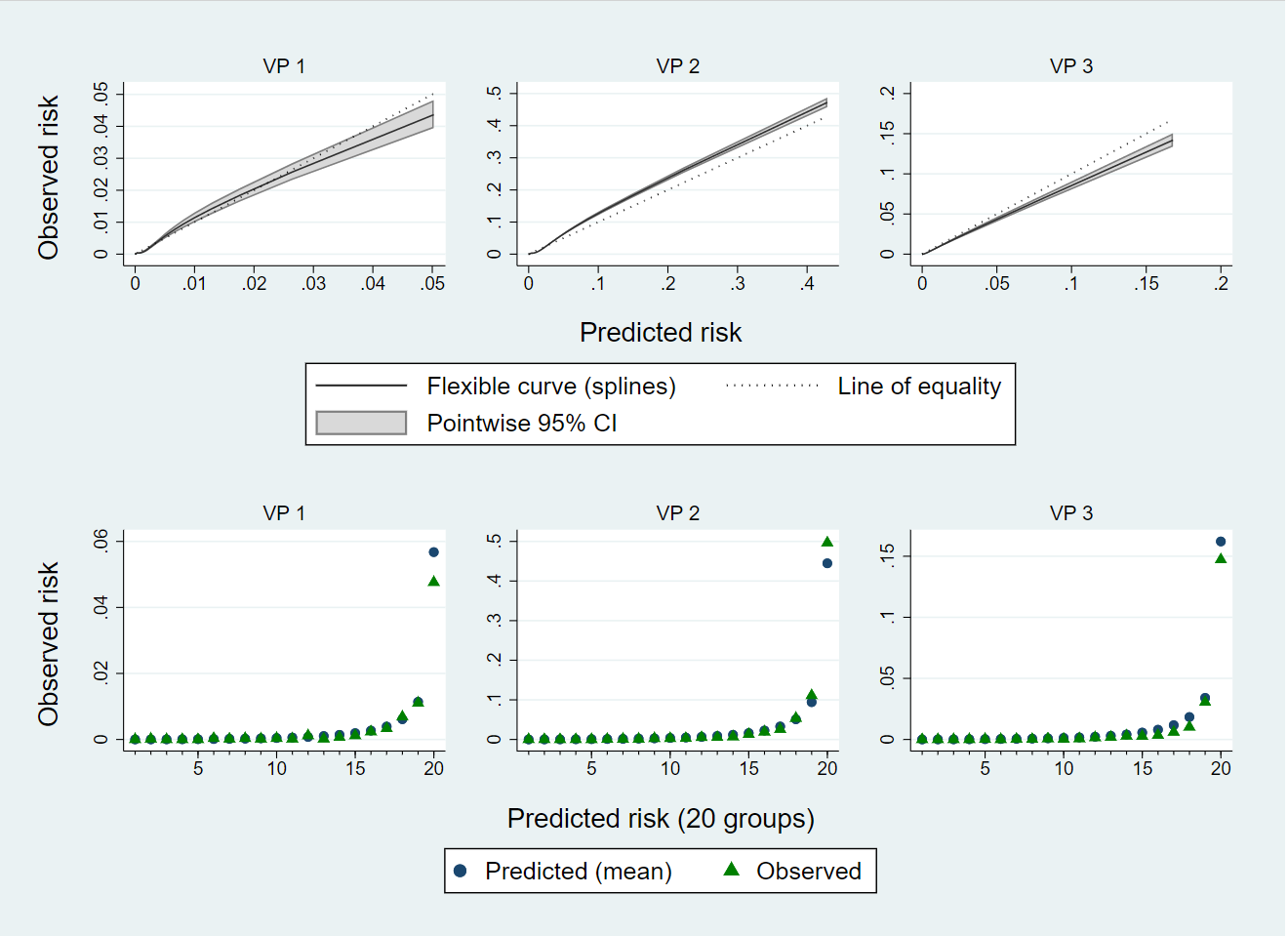


Figure S5: Approach B “selected” model including A&E COVID-19 attendances – flexible calibration curves (top) and observed vs predicted COVID-19 mortality by twentieths of predicted risk for each validation period (VP 1, 2 and 3).


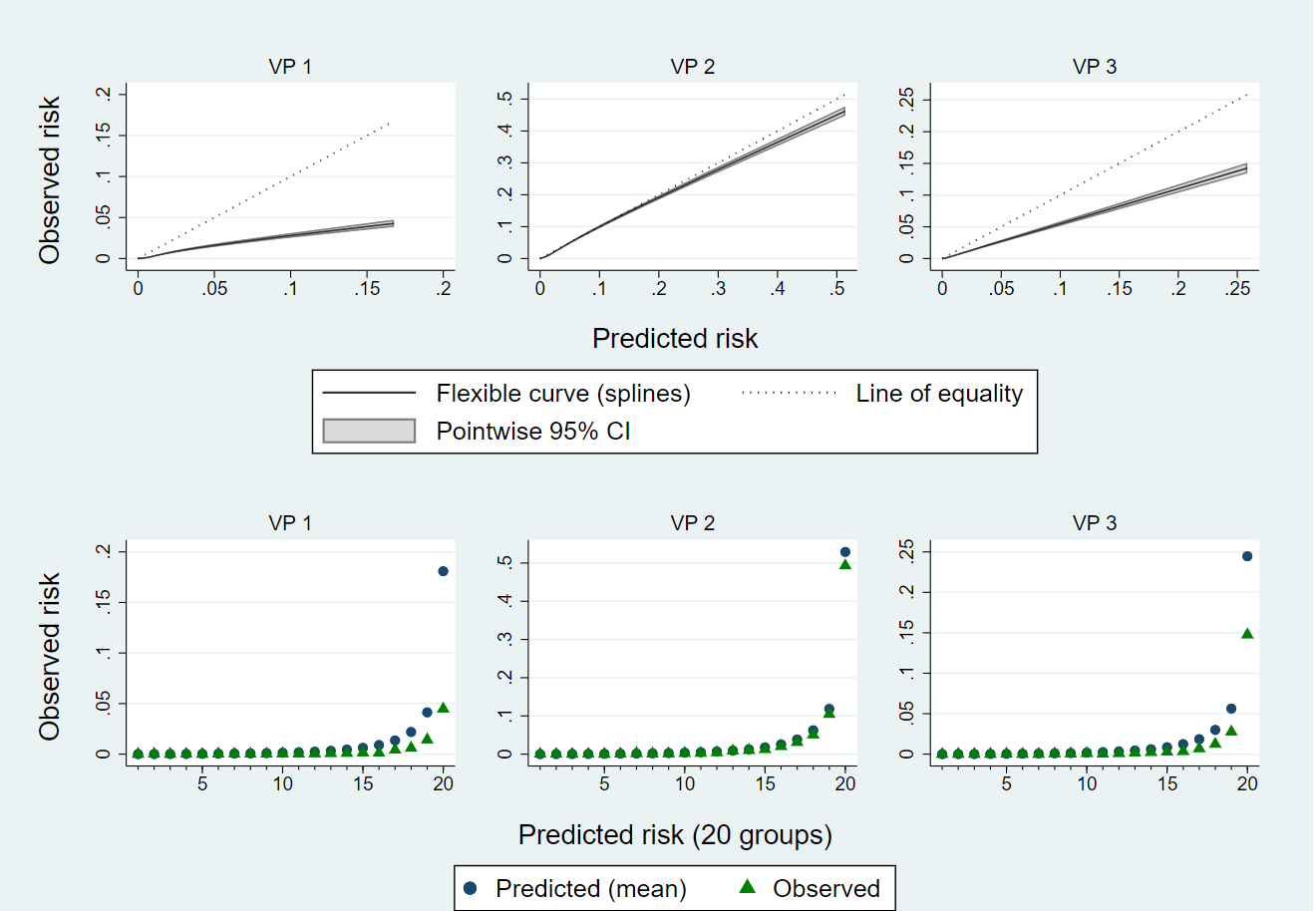


Figure S6: Approach B “selected” model including suspected COVID-19 in primary care – flexible calibration curves (top) and observed vs predicted COVID-19 mortality by twentieths of predicted risk for each validation period (VP 1, 2 and 3).


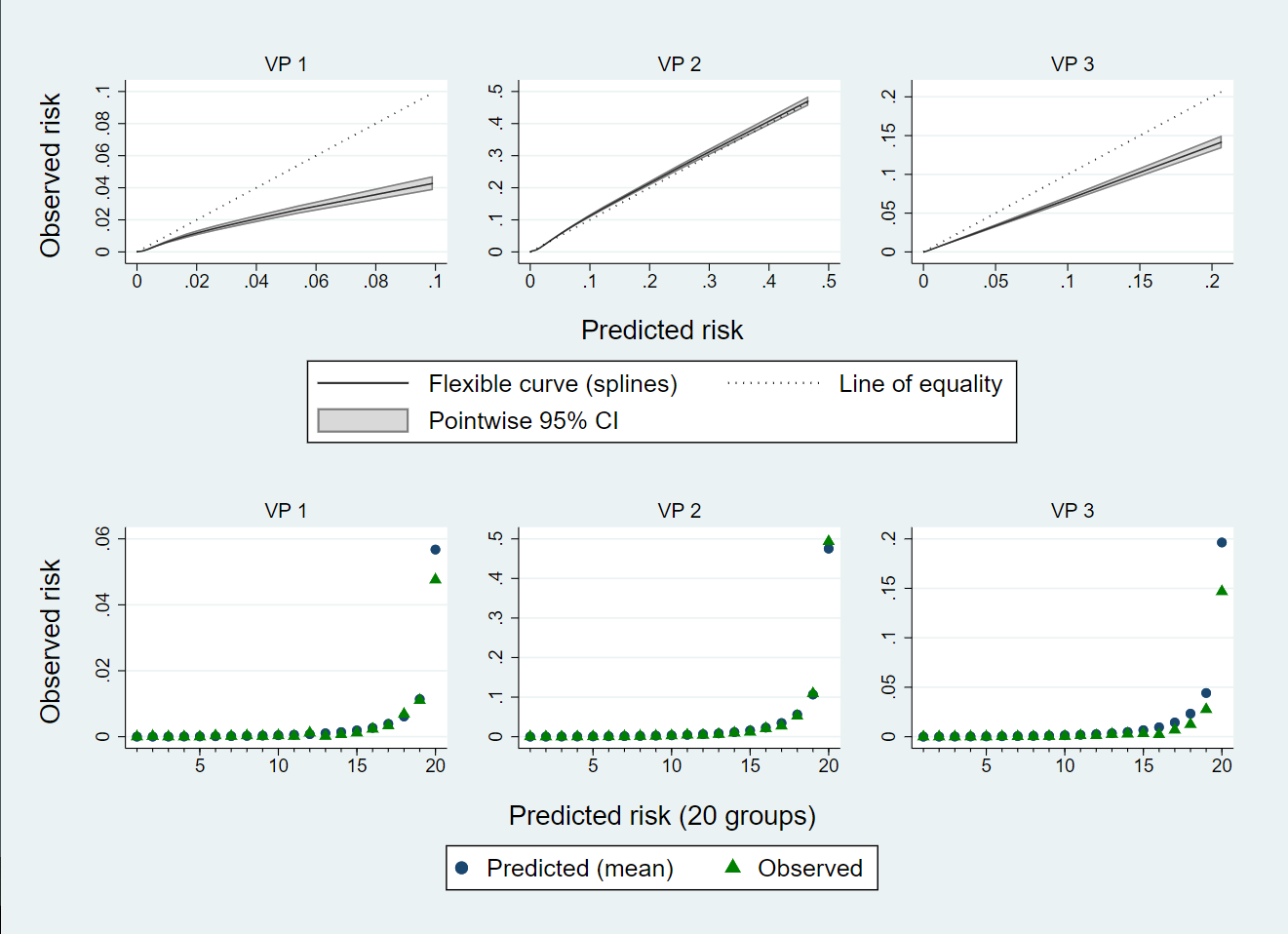


# Geographical and temporal internal validation results

The results in this section are an additional type of internal validation named “internal-external” by Steyerberg et al^3^. These validations are intended to assess the performance of the models in geographical and temporal contexts not used in the data fitting. However, this is not external validation, since the original data are used to perform this validation.

Table S6 shows measures of model performance for temporal validation for approach B using A&E attendance and primary care data as proxy measures of infection prevalence. Table S7 shows measures of model performance for geographical internal validation for the same proxy measures. Results for approach A and approach B using modelled estimates are provided in the main text.

Figures S7-S9 show measures of model performance for the geographical and temporal internal-external validation.

Table S6. Measures of model performance for temporal validation for the approach B “selected” model using A&E attendance and primary care data as proxy measures of infection prevalence

| Measures of infection prevalence included (approach) and model form | Validation  period | C-statistic | Observed  mean risk  (%) | Predicted  mean risk  (%) | Calibration | |
| --- | --- | --- | --- | --- | --- | --- |
|  |  |  |  |  | Intercept  (95% CI) | Slope  (95% CI) |
| A&E COVID-19 attendances (B),  Poisson | 3 | 0.941 | 0.0104 | 0.0297 | -1.05  (-1.11, -1.00) | 1.04  (0.99, 1.10) |
| Suspected COVID-19 in primary care (B), Poisson | 3 | 0.941 | 0.0104 | 0.0241 | -0.84  (-0.90, -0.79) | 1.05  (0.99, 1.10) |

Table S7. Measures of model performance for geographical validation for the approach B “selected model” using A&E attendance and primary care data as proxy measures of infection prevalence

| Measures of infection prevalence included (approach) and model form | Validation period | Region | C-statistic | Observed mean risk (%) | Predicted mean risk (%) | Calibration | | |
| --- | --- | --- | --- | --- | --- | --- | --- | --- |
|  |  |  |  |  |  | Intercept (95% CI) | Slope (95% CI) |  |
| A&E COVID-19 attendances (B),  Poisson | 1 | East | 0.912 | 0.0036 | 0.0129 | -1.29 (-1.49, -1.09) | 0.88 (0.68, 1.08) |  |
|  |  | London | 0.950 | 0.0102 | 0.0105 | -0.03 (-0.22, 0.16) | 1.00 (0.80, 1.19) |  |
|  |  | Midlands | 0.924 | 0.0052 | 0.0148 | -1.06 (-1.23, -0.89) | 0.93 (0.76, 1.10) |  |
|  |  | North East/Yorks | 0.909 | 0.0024 | 0.0156 | -1.88 (-2.14, -1.61) | 0.83 (0.57, 1.10) |  |
|  |  | North West | 0.843 | 0.0021 | 0.0163 | -2.06 (-2.48, -1.64) | 0.68 (0.26, 1.10) |  |
|  |  | South East | 0.947 | 0.0025 | 0.0135 | -1.67 (-2.12, -1.22) | 1.05 (0.60, 1.50) |  |
|  |  | South West | 0.936 | 0.0014 | 0.0167 | -2.49 (-2.92, -2.06) | 0.96 (0.54, 1.39) |  |
|  | 2 | East | 0.933 | 0.0365 | 0.0420 | -0.14 (-0.20, -0.08) | 1.04 (0.98, 1.10) |  |
|  |  | London | 0.934 | 0.0396 | 0.0223 | 0.58 (0.48, 0.68) | 0.91 (0.81, 1.01) |  |
|  |  | Midlands | 0.931 | 0.0405 | 0.0618 | -0.43 (-0.49, -0.37) | 0.96 (0.90, 1.02) |  |
|  |  | North East/Yorks | 0.935 | 0.0420 | 0.0377 | 0.11 (0.05, 0.17) | 0.98 (0.91, 1.04) |  |
|  |  | North West | 0.927 | 0.0493 | 0.0544 | -0.10 (-0.19, -0.01) | 0.98 (0.90, 1.07) |  |
|  |  | South East | 0.939 | 0.0238 | 0.0261 | -0.09 (-0.24, 0.05) | 1.06 (0.91, 1.21) |  |
|  |  | South West | 0.940 | 0.0235 | 0.0363 | -0.44 (-0.54, -0.33) | 1.05 (0.94, 1.15) |  |
|  | 3 | East | 0.943 | 0.0106 | 0.0187 | -0.57 (-0.68, -0.45) | 1.07 (0.95, 1.18) |  |
|  |  | London | 0.948 | 0.0033 | 0.0134 | -1.39 (-1.73, -1.05) | 0.94 (0.60, 1.28) |  |
|  |  | Midlands | 0.938 | 0.0120 | 0.0224 | -0.63 (-0.74, -0.52) | 1.01 (0.90, 1.12) |  |
|  |  | North East/Yorks | 0.944 | 0.0142 | 0.0227 | -0.48 (-0.59, -0.36) | 1.02 (0.91, 1.13) |  |
|  |  | North West | 0.936 | 0.0168 | 0.0208 | -0.22 (-0.36, -0.07) | 1.03 (0.88, 1.18) |  |
|  |  | South East | 0.966 | 0.0052 | 0.0126 | -0.88 (-1.19, -0.56) | 1.19 (0.87, 1.50) |  |
|  |  | South West | 0.927 | 0.0047 | 0.0203 | -1.46 (-1.70, -1.23) | 1.08 (0.85, 1.31) |  |

Table S7 ctd. Measures of model performance for geographical validation for the approach B “selected model” using A&E attendance and primary care data as proxy measures of infection prevalence

| Measures of infection prevalence included (approach) and model form | Validation period | Region | C-statistic | Observed mean risk (%) | Predicted mean risk (%) | Calibration | | |
| --- | --- | --- | --- | --- | --- | --- | --- | --- |
|  |  |  |  |  |  | Intercept (95% CI) | Slope (95% CI) |  |
| Suspected COVID-19 in primary care (B), Poisson | 1 | East | 0.912 | 0.0036 | 0.0088 | -0.91 (-1.11, -0.71) | 0.87 (0.67, 1.07) |  |
|  |  | London | 0.953 | 0.0102 | 0.0066 | 0.44 (0.25, 0.63) | 1.00 (0.81, 1.20) |  |
|  |  | Midlands | 0.916 | 0.0052 | 0.0079 | -0.42 (-0.59, -0.26) | 0.88 (0.71, 1.04) |  |
|  |  | North East/Yorks | 0.911 | 0.0024 | 0.0095 | -1.37 (-1.64, -1.11) | 0.83 (0.57, 1.10) |  |
|  |  | North West | 0.857 | 0.0021 | 0.0047 | -0.80 (-1.22, -0.38) | 0.70 (0.29, 1.12) |  |
|  |  | South East | 0.936 | 0.0025 | 0.0096 | -1.33 (-1.78, -0.88) | 1.00 (0.55, 1.45) |  |
|  |  | South West | 0.932 | 0.0014 | 0.0102 | -1.99 (-2.42, -1.57) | 0.93 (0.50, 1.35) |  |
|  | 2 | East | 0.935 | 0.0365 | 0.0312 | 0.16 (0.10, 0.22) | 1.02 (0.96, 1.09) |  |
|  |  | London | 0.936 | 0.0396 | 0.0274 | 0.37 (0.27, 0.47) | 0.92 (0.82, 1.02) |  |
|  |  | Midlands | 0.933 | 0.0405 | 0.0396 | 0.02 (-0.04, 0.08) | 0.99 (0.93, 1.05) |  |
|  |  | North East/Yorks | 0.938 | 0.0420 | 0.0423 | -0.01 (-0.07, 0.06) | 0.99 (0.92, 1.05) |  |
|  |  | North West | 0.927 | 0.0493 | 0.0402 | 0.21 (0.12, 0.29) | 0.97 (0.89, 1.06) |  |
|  |  | South East | 0.942 | 0.0238 | 0.0367 | -0.44 (-0.58, -0.29) | 1.04 (0.89, 1.19) |  |
|  |  | South West | 0.940 | 0.0235 | 0.0429 | -0.61 (-0.71, -0.50) | 1.02 (0.91, 1.12) |  |
|  | 3 | East | 0.942 | 0.0106 | 0.0158 | -0.40 (-0.52, -0.28) | 1.05 (0.93, 1.16) |  |
|  |  | London | 0.951 | 0.0033 | 0.0076 | -0.82 (-1.16, -0.48) | 0.93 (0.59, 1.27) |  |
|  |  | Midlands | 0.937 | 0.0120 | 0.0167 | -0.33 (-0.44, -0.22) | 0.99 (0.88, 1.10) |  |
|  |  | North East/Yorks | 0.943 | 0.0142 | 0.0179 | -0.23 (-0.34, -0.12) | 1.03 (0.92, 1.14) |  |
|  |  | North West | 0.936 | 0.0168 | 0.0144 | 0.15 (0.00, 0.30) | 1.04 (0.89, 1.19) |  |
|  |  | South East | 0.963 | 0.0052 | 0.0160 | -1.12 (-1.43, -0.80) | 1.14 (0.82, 1.45) |  |
|  |  | South West | 0.921 | 0.0047 | 0.0161 | -1.23 (-1.46, -1.00) | 1.05 (0.82, 1.28) |  |

Figure S7. Measures of model performance for the “selected” models in the internal validation over the three validation periods. Dotted lines represent the ideal calibration slope (1) and intercept (0). For overall mean prediction, dashed lines represent the observed mean in the relevant validation cohort. Graph titles show approach: ME modelled estimates, AE A&E COVID-19 attendances, GP suspected COVID-19 in primary care.

Figure S8. Measures of model performance for the “selected” models in the temporal validation, undertaken only in the third validation period. Dotted lines represent the ideal calibration slope (1) and intercept (0). For overall mean prediction, dashed lines represent the observed mean in the third validation cohort. Graph titles show the approach: ME modelled estimates, AE A&E COVID-19 attendances, GP suspected COVID-19 in primary care.

Figure S9. Measures of model performance for the “selected” models in the geographical validation. Clusters of points of the same colour show, from left to right, models omitting regions: East, London, Midlands, North East and Yorkshire, North West, South East and South West, respectively). Dotted lines represent the ideal calibration slope (1) and intercept (0). Graph titles show the approach: ME modelled estimates, AE A&E COVID-19 attendances, GP suspected COVID-19 in primary care.

# Protocol deviations

We made a few minor deviations from the protocol, relating to the variable selection.^4^ Our pre-published plan involved using a 4% random sample for variable selection via the lasso, to reduce computational time. For the geographical internal validation, a 4% random sample led, in some cases, to an insufficiently small sample size. Therefore, the sampling fraction was increased to result in approximately the same sample size as was used for the main analysis (5% when omitting region 1 or regions 4-7 and 7% when omitting the larger regions 2 and 3).

# Additional sensitivity analysis results

## Comparing the cohort and case-cohort design

Table S8 shows the model coefficients from the Cox model fitted in the case-cohort sample, using the “selected” predictor set, and the coefficients from fitting a model with the same predictors but in the whole cohort. The estimated coefficients are, as expected, very similar.

Table S9 shows measures of model performance of the case-cohort model and the full cohort model, each validated in the three validation cohorts. The predictive performance of the two models is very similar.

These tables provide reassurance that differences between approaches and models are unlikely to be affected by the case-cohort design used in the model development stage.

## Additional model types

Results from replacing the Cox model with a Royston-Parmar, or Generalised gamma model are not shown. Results from a Weibull model are discussed below. Broadly, the Royston-Parmar models had very similar performance to the Cox models but the Weibull and Gamma models generally had poorer calibration.

Results from replacing the Poisson model in approach B with a logistic model or Weibull model showed that performance was very similar to that of the Poisson.

Table S10 shows measures of model performance, using a Weibull model in place of the Cox model for approach A and replacing the Poisson model by a Weibull model for approach B (including modelled estimates of infection prevalence). Thus Table S10 compares the approaches A and B using the same model format, removing the difference in model format type from the comparison. The overall message is the same as in the main results. The calibration of approach A, which for the Cox model was good in validation period 1 but poor in periods 2 and 3, is poor in all three periods using the Weibull model. The Weibull model is overly restrictive here, resulting in poorer calibration in the first validation period. However, Table S10 suggests that differences between approach A and B observed in the main text are not due to the differences in using a Cox model for approach A and a Poisson model for approach B.

## Combining proxy measures of the burden of COVID-19 infection

Models including all three proxies of infection had similar performance to the models including only modelled estimates, which had the best measures of performance. Including both the A&E COVID-19 rates and suspected case rates in primary care fractionally improved model performance compared to the two models singly including those proxies.

## Not updating patient characteristics in landmark sub-studies

In models in which patient characteristics were not updated over time for approach B, as expected, model performance was very similar to the main models, because the larger portion of patient characteristics stayed the same through the 100 day period included.

## Removing the proxies of burden of infection

When using approach B to predict COVID-19 infection but omitting the proxy measures of infection prevalence, the mean predicted risk, was the same in each of the three validation periods, as it was for approach A. Therefore, these models fail to distinguish between periods with higher and lower risk. However, the mean predicted risk was higher than for approach A because these models essentially average that risk over all possible 28 day periods, in contrast to approach A which was estimating for the first 28 days in the cohort.

Therefore, landmark models without the proxies for burden of infection, retained high discrimination but had poor calibration, similar to approach A models.

Table S8. Estimated Hazard Ratios from a Cox model, approach A for the “selected” model, comparing a model fitted in the case-cohort sample and one fitted in the whole cohort

| Characteristic | Sub-distribution Hazard Ratio (95% CI)  **Case-cohort** | Sub-distribution Hazard Ratio (95% CI)  **Full cohort** |
| --- | --- | --- |
| Age (per unit*) | 7.56 (7.16, 7.98) | 7.49 (7.11,7.89) |
| Male | 2.13 (1.97, 2.29) | 2.14 (1.99, 2.30) |
| Urban | 1.52 (1.42, 1.62) | 1.52 (1.42, 1.61) |
| IMD 1 (least deprived) | 0.79 (0.74, 0.84) | 0.79 (0.74, 0.84) |
| IMD 5 (most deprived) | 1.33 (1.22, 1.45) | 1.31 (1.21, 1.42) |
| Ethnicity: White | 0.58 (0.54, 0.63) | 0.58 (0.54, 0.62) |
| BMI: Underweight | 1.53 (1.33, 1.75) | 1.51 (1.33, 1.71) |
| BMI: Normal/overweight | 0.81 (0.77, 0.86) | 0.82 (0.78, 0.87) |
| Diabetes: None | 0.68 (0.64, 0.72) | 0.68 (0.64, 0.72) |
| Diabetes: HbA1c unknown | 1.22 (1.07, 1.39) | 1.26 (1.12, 1.42) |
| No stroke | 0.71 (0.64, 0.79) | 0.71 (0.65, 0.79) |
| No dementia | 0.36 (0.30, 0.42) | 0.33 (0.29, 0.39) |
| No other neurological condition | 0.14 (0.10, 0.18) | 0.14 (0.11, 0.18) |
| Asthma: With OCS use | 1.23 (1.06, 1.43) | 1.32 (1.16, 1.51) |
| No respiratory disease | 0.35 (0.28, 0.42) | 0.36 (0.30, 0.43) |
| Cancer (exc. haematological): Last year | 1.63 (1.34, 2.00) | 1.76 (1.46, 2.13) |
| Cancer (haematological): Never | 0.44 (0.37, 0.53) | 0.43 (0.37, 0.50) |
| No liver disease | 0.27 (0.17, 0.41) | 0.25 (0.18, 0.35) |
| Renal impairment: None | 0.70 (0.65, 0.76) | 0.70 (0.65, 0.76) |
| Renal impairment: Stage 4/5 | 10.08 (7.24, 14.03) | 11.23 (8.60, 14.66) |
| No immunosuppression | 0.47 (0.30, 0.74) | 0.51 (0.35, 0.74) |
| No serious mental illness | 0.41 (0.36, 0.48) | 0.42 (0.37, 0.47) |
| *Interactions with age***:* |  |  |
| BMI: Obese II (Per unit age increase) | 1.14 (1.06, 1.21) | 1.13 (1.06, 1.20) |
| BMI: Obese III (Per unit age increase) | 0.94 (0.79, 1.12) | 0.97 (0.82, 1.14) |
| Current smoker (Per unit age increase) | 0.97 (0.91, 1.03) | 0.99 (0.93, 1.04) |
| Diagnosed hypertension (Per unit age increase) | 0.98 (0.95, 1.01) | 0.98 (0.95, 1.01) |
| Diabetes: Uncontrolled (Per unit age increase) | 0.99 (0.92, 1.07) | 1.01 (0.94, 1.08) |
| Other neurological condition (Per unit age increase) | 0.51 (0.43, 0.61) | 0.53 (0.45, 0.61) |
| Respiratory disease (Per unit age increase) | 0.70 (0.62, 0.78) | 0.71 (0.64, 0.79) |
| Cancer (haematological): Last year (Per unit age increase) | 1.07 (0.78, 1.46) | 1.09 (0.82, 1.45) |
| Liver disease (Per unit age increase) | 0.62 (0.46, 0.85) | 0.57 (0.45, 0.73) |
| Dialysis (Per unit age increase) | 1.45 (1.17, 1.80) | 1.26 (1.06, 1.50) |
| Renal impairment: Stage 4/5 (Per unit age increase) | 0.37 (0.31, 0.44) | 0.35 (0.31, 0.40) |
| Asplenia (Per unit age increase) | 1.01 (0.77, 1.33) | 0.99 (0.77, 1.26) |
| Inflammatory bowel disease (Per unit age increase) | 1.11 (1.00, 1.23) | 1.11 (1.01, 1.21) |
| Fracture (Per unit age increase) | 1.25 (1.16, 1.34) | 1.25 (1.17, 1.34) |
| *Interactions with sex:* |  |  |
| (In males) IMD 5 (most deprived) | 0.97 (0.86, 1.08) | 0.97 (0.87, 1.08) |
| (In females) BMI: Obese III | 1.83 (1.36, 2.48) | 1.80 (1.36, 2.39) |
| (In males) Diabetes: Uncontrolled | 1.39 (1.19, 1.62) | 1.36 (1.18, 1.56) |
| (In females) Cardiac disease | 1.30 (1.19, 1.41) | 1.31 (1.21, 1.42) |
| (In females) Atrial Fibrillation | 1.22 (1.11, 1.34) | 1.20 (1.09, 1.31) |
| (In males) Surgery for peripheral arterial disease | 1.72 (1.41, 2.09) | 1.71 (1.45, 2.02) |
| (In males) Stroke | 1.05 (0.91, 1.21) | 1.05 (0.93, 1.20) |
| (In males) Dementia | 1.22 (0.97, 1.54) | 1.10 (0.91, 1.34) |
| (In females) Asthma: Without OCS use | 1.07 (0.96, 1.18) | 1.07 (0.98, 1.18) |
| (In females) Respiratory disease | 1.26 (1.12, 1.42) | 1.24 (1.11, 1.38) |
| (In females) Cancer (exc. haematological): Last year | 2.01 (1.49, 2.72) | 1.75 (1.33, 2.31) |
| (In males) Cancer (exc. haematological): 2-5 years ago | 1.25 (1.09, 1.44) | 1.24 (1.09, 1.41) |
| (In males) Cancer (exc. haematological): 5+ years ago | 1.06 (0.96, 1.17) | 1.07 (0.98, 1.18) |
| (In females) Cancer (haematological): Last year | 1.28 (0.54, 3.02) | 1.22 (0.55, 2.67) |
| (In males) Cancer (haematological): 5+ years ago | 0.60 (0.44, 0.82) | 0.55 (0.41, 0.74) |
| (In females) Liver disease | 1.27 (0.88, 1.82) | 1.43 (1.06, 1.93) |
| (In females) Dialysis | 1.61 (0.98, 2.63) | 1.75 (1.18, 2.58) |
| (In females) Renal impairment: Stage 4/5 | 1.25 (1.02, 1.54) | 1.23 (1.02, 1.48) |
| (In males) Renal impairment: Stage 3a/3b | 0.96 (0.86, 1.07) | 0.95 (0.86, 1.04) |
| (In males) RA/SLE/Psoriasis | 1.12 (1.00, 1.26) | 1.12 (1.00, 1.24) |
| (In males) Fracture | 1.65 (1.32, 2.06) | 1.56 (1.28, 1.89) |
|  | Estimated baseline survival | Estimated baseline survival |
| At 28 days | 0.9873567 | 0.97765011 |

* Age measured in SD units (1 unit = 18.84 years).

Abbreviations: BMI Body Mass Index; IMD Index of Multiple Deprivation; OCS Oral corticosteroids; RA rheumatoid arthritis; SLE Systemic lupus erythematosus.

Table S9. Measures of model performance in predicting 28-day risk of COVID-19 mortality using the “selected” models, comparing results from the case-cohort approach to those obtained from developing the model in the full cohort

| Approach | Measures of infection prevalence included (approach), model | Validation  period | C-statistic | Observed mean risk (%) | Predicted mean risk (%) | Calibration | |
| --- | --- | --- | --- | --- | --- | --- | --- |
|  |  |  |  |  |  | Intercept (95% CI) | Slope (95% CI) |
| Case-cohort (main analysis) | None (A), Cox | 1 | 0.924 | 0.0038 | 0.0038 | 0.00 (-0.10, 0.09) | 0.95 (0.86, 1.05) |
|  |  | 2 | 0.934 | 0.0374 | 0.0038 | 2.30 (2.27, 2.32) | 1.02 (0.99, 1.05) |
|  |  | 3 | 0.941 | 0.0104 | 0.0037 | 1.03 (0.97, 1.08) | 1.05 (1.00, 1.11) |
| Full cohort | None (A), Cox | 1 | 0.924 | 0.0038 | 0.0038 | 0.00 (-0.09, 0.09) | 0.96 (0.87, 1.05) |
|  |  | 2 | 0.934 | 0.0374 | 0.0038 | 2.30 (2.27, 2.33) | 1.02 (0.99, 1.05) |
|  |  | 3 | 0.941 | 0.0104 | 0.0037 | 1.03 (0.98, 1.09) | 1.06 (1.00, 1.11) |

Table S10. Measures of model performance in predicting 28-day risk of COVID-19 mortality using the “selected” models, comparing results from approach A and B using a Weibull model for each (in contrast to the Cox (A) and Poisson (B) models used in the main comparison)

| Measures of infection prevalence included (approach), model | Validation  period | C-statistic | Observed mean risk (%) | Predicted mean risk (%) | Calibration | |
| --- | --- | --- | --- | --- | --- | --- |
|  |  |  |  |  | Intercept (95% CI) | Slope (95% CI) |
| None (A), Weibull | 1 | 0.924 | 0.0038 | 0.0111 | -1.08 (-1.17, -0.98) | 0.96 (0.87, 1.05) |
|  | 2 | 0.934 | 0.0374 | 0.0110 | 1.22 (1.20, 1.25) | 1.02 (0.99, 1.05) |
|  | 3 | 0.941 | 0.0104 | 0.0109 | -0.04 (-0.10, 0.01) | 1.06 (1.00, 1.11) |
| Modelled estimates (B), Weibull | 1 | 0.925 | 0.0038 | 0.0044 | -0.15 (-0.24, -0.06) | 0.93 (0.84, 1.02) |
|  | 2 | 0.937 | 0.0374 | 0.0354 | 0.06 (0.03, 0.09) | 1.00 (0.97, 1.03) |
|  | 3 | 0.944 | 0.0104 | 0.0128 | -0.20 (-0.26, -0.15) | 1.03 (0.97, 1.09) |

# Third approach: daily landmarking

A third statistical approach was detailed in our pre-published protocol. This is not presented in the main text, but for reasons of transparency we wished to present all results discussed in the protocol. The results from this approach are therefore presented below.

In addition to the two approaches in the main text, a third approach was used: (C) daily landmarking. In comparison to the landmarking approach B, this approach additionally updates the measures of the infection prevalence throughout the 28-day period, to try to better estimate the relationship between current infection prevalence and risk.

## Design

Approach C also used a series of stacked sub-studies, with each lasting a single day. Thus 100 sub-studies were formed, the first starting on 1^st^ March 2020 and the last starting on 8^th^ June 2020. Each sub-study included all cases (COVID-19 related deaths) occurring on that day and a random age-stratified sample of non-cases who remained alive by the previous day, with sampling fractions equal to 1/100 of the sampling fractions for approach A. The outcome was the binary outcome of whether or not the sub-study participant experienced a COVID-19 related death on that day. This approach also required information about the daily rate of death due to other causes, which was estimated in a second case-cohort sample, comprising a sampling fraction of 0.3 of all non-COVID-19 related deaths on each day and an age-stratified sample of participants who did not die of non-COVID-19 related causes on that day, with sampling fractions equal to 1/100 of the sampling fractions for approach A.

## Statistical analysis

For approach C, the series of 1-day studies were stacked to form one analysis dataset. A Poisson model was fitted to estimate the daily rate of COVID-19 related death using inverse sampling weights with robust standard errors, incorporating predictors selected by the lasso and proxy measures of the burden of infection. A similar Poisson model was fitted to estimate the daily rate of mortality due to non-COVID-19-related causes conditional on the same set of predictor variables, but without the measures of the burden of infection, weighted according to the inverse of the sampling fractions. Risk of 28-day COVID-19 related death was estimated by combining the estimates of daily survival from COVID-19 related death and other causes.

To predict risk over a 28-day period, estimates of the burden of infection throughout the 28 day period are required. Model performance was therefore undertaken in a number of ways. First, the 28-day evolution of proxy measures were assumed known. Second, the measure was taken at day 0 of the 28-day period and assumed constant for the next 28 days. Third, the measures prior to day 1 of the 28 day period were used to predict values over the 28 day period using fractional polynomials.

## Results

Approach C models performed similarly to approach B when using the actual measures of infection prevalence through the 28 day period. However, poor estimation of the measure using only data available at day 0 of the period led, in some cases, to large under- and over-estimation of risks (Table S11).

Table S11. Measures of model performance using approach C, daily landmarking. Proxy measures of the burden of infection are required through the 28 day validation period. These are obtained in three ways: (i) the actual measure as it evolves (which would not be available in practice), (ii) assuming the measure remains constant from the beginning of the validation period, and (iii) using a fractional polynomial model fitted to the prior 3 weeks of data.

| Burden of infection proxy measure used | Validation period | C-statistic | Observed mean risk (%) | Predicted mean risk (%) | Estimated calibration intercept  (95% CI) | Estimated calibration slope  (95% CI) |
| --- | --- | --- | --- | --- | --- | --- |
| **Actual measure** **obtained through 28 day validation period** | | | | | | |
| Modelled estimates | 1 | 0.926 | 0.0038 | 0.0064 | -0.52 (-0.62, -0.43) | 0.91 (0.81, 1.00) |
|  | 2 | 0.936 | 0.0374 | 0.0333 | 0.12 (0.09, 0.15) | 0.98 (0.96, 1.01) |
|  | 3 | 0.944 | 0.0104 | 0.0108 | -0.03 (-0.09, 0.02) | 1.03 (0.97, 1.08) |
| A&E COVID-19 attendance rates | 1 | 0.928 | 0.0038 | 0.0065 | -0.53 (-0.62, -0.44) | 0.95 (0.85, 1.04) |
|  | 2 | 0.932 | 0.0374 | 0.0347 | 0.08 (0.05, 0.10) | 0.98 (0.95, 1.01) |
|  | 3 | 0.942 | 0.0104 | 0.0113 | -0.08 (-0.13, -0.02) | 0.99 (0.93, 1.05) |
| Suspected COVID-19 case rates in primary care | 1 | 0.923 | 0.0038 | 0.0047 | -0.21 (-0.31, -0.12) | 0.93 (0.83, 1.02) |
|  | 2 | 0.933 | 0.0374 | 0.0328 | 0.13 (0.10, 0.16) | 0.98 (0.95, 1.01) |
|  | 3 | 0.940 | 0.0104 | 0.0142 | -0.31 (-0.37, -0.26) | 1.01 (0.96, 1.07) |
| **Assuming measure remains constant from day 0 of validation period** | | | | | | |
| Modelled estimates | 1 | 0.924 | 0.0038 | 0.0014 | 1.03 (0.94, 1.12) | 0.90 (0.81, 0.99) |
|  | 2 | 0.937 | 0.0374 | 0.0316 | 0.17 (0.14, 0.20) | 0.99 (0.96, 1.01) |
|  | 3 | 0.944 | 0.0104 | 0.0107 | -0.03 (-0.08, 0.03) | 1.02 (0.97, 1.08) |
| A&E COVID-19 attendance rates | 1 | 0.922 | 0.0038 | 0.0018 | 0.78 (0.68, 0.87) | 0.92 (0.83, 1.01) |
|  | 2 | 0.932 | 0.0374 | 0.0439 | -0.16 (-0.19, -0.13) | 0.98 (0.95, 1.01) |
|  | 3 | 0.942 | 0.0104 | 0.0160 | -0.43 (-0.49, -0.38) | 1.00 (0.95, 1.06) |
| Suspected COVID-19 case rates in primary care | 1 | 0.920 | 0.0038 | 0.0006 | 1.86 (1.77, 1.95) | 0.91 (0.81, 1.00) |
|  | 2 | 0.932 | 0.0374 | 0.0485 | -0.26 (-0.29, -0.23) | 0.98 (0.95, 1.00) |
|  | 3 | 0.940 | 0.0104 | 0.0177 | -0.53 (-0.59, -0.48) | 1.02 (0.97, 1.08) |
| **Predicted measure based on fractional polynomial model of previous 3 weeks of data** | | | | | | |
| Modelled estimates | 1 | 0.926 | 0.0038 | 0.0040 | -0.06 (-0.15, 0.03) | 0.91 (0.82, 1.00) |
|  | 2 | 0.632 | 0.0374 | 13.392 | * | * |
|  | 3 | 0.828 | 0.0104 | 3.3729 | * | * |
| A&E COVID-19 attendance rates | 1 | 0.922 | 0.0038 | 0.0018 | 0.78 (0.68, 0.87) | 0.92 (0.83, 1.01) |
|  | 2 | 0.921 | 0.0374 | 0.0392 | -0.05 (-0.08, -0.02) | 0.77 (0.74, 0.80) |
|  | 3 | 0.936 | 0.0104 | 0.02242 | -0.77 (-0.83, -0.71) | 0.94 (0.88, 0.99) |
| Suspected COVID-19 case rates in primary care | 1 | 0.919 | 0.0038 | 0.00315 | 0.19 (0.09, 0.28) | 0.90 (0.8, 0.99) |
|  | 2 | 0.901 | 0.0374 | 0.10474 | -1.06 (-1.09, -1.03) | 0.77 (0.74, 0.80) |
|  | 3 | 0.931 | 0.0104 | 0.00909 | 0.14 (0.08, 0.19) | 0.93 (0.87, 0.98) |

* Unable to be estimated (due to huge overestimation of risk overall)

# References

1. Davies NG, Kucharski AJ, Eggo RM, et al. Effects of non-pharmaceutical interventions on COVID-19 cases, deaths, and demand for hospital services in the UK: a modelling study. *Lancet Public Health*. 2020;5(7):e375-e385. doi:10.1016/S2468-2667(20)30133-X

2. Williamson EJ, Walker AJ, Bhaskaran K, et al. Factors associated with COVID-19-related death using OpenSAFELY. *Nature*. 2020;584(7821):430-436. doi:10.1038/s41586-020-2521-4

3. Steyerberg EW, Harrell FE Jr. Prediction models need appropriate internal, internal-external, and external validation. *J Clin Epidemiol*. 2016;69:245-247. doi:10.1016/j.jclinepi.2015.04.005

4. Williamson E, Tazare J, Bhaskaran K, et al. Study protocol: Comparison of different risk prediction modelling approaches for COVID-19 related death using the OpenSAFELY platform [version 1; peer review: 1 approved]. *Wellcome Open Res*. 2020;5(243). doi:10.12688/wellcomeopenres.16353.1
